# Phosphorylation of the nuclear poly(A) binding protein (PABPN1) during mitosis protects mRNA from hyperadenylation and maintains transcriptome dynamics

**DOI:** 10.1093/nar/gkae562

**Published:** 2024-06-29

**Authors:** Jackson M Gordon, David V Phizicky, Leonard Schärfen, Courtney L Brown, Dahyana Arias Escayola, Jean Kanyo, TuKiet T Lam, Matthew D Simon, Karla M Neugebauer

**Affiliations:** Department of Molecular Biophysics and Biochemistry, Yale University, New Haven, CT 06520, USA; Department of Molecular Biophysics and Biochemistry, Yale University, New Haven, CT 06520, USA; Department of Molecular Biophysics and Biochemistry, Yale University, New Haven, CT 06520, USA; Department of Molecular Biophysics and Biochemistry, Yale University, New Haven, CT 06520, USA; Department of Molecular Biophysics and Biochemistry, Yale University, New Haven, CT 06520, USA; Keck MS & Proteomics Resource, Yale School of Medicine, New Haven, CT 06520, USA; Department of Molecular Biophysics and Biochemistry, Yale University, New Haven, CT 06520, USA; Keck MS & Proteomics Resource, Yale School of Medicine, New Haven, CT 06520, USA; Department of Molecular Biophysics and Biochemistry, Yale University, New Haven, CT 06520, USA; Department of Molecular Biophysics and Biochemistry, Yale University, New Haven, CT 06520, USA

## Abstract

Polyadenylation controls mRNA biogenesis, nucleo-cytoplasmic export, translation and decay. These processes are interdependent and coordinately regulated by poly(A)-binding proteins (PABPs), yet how PABPs are themselves regulated is not fully understood. Here, we report the discovery that human nuclear PABPN1 is phosphorylated by mitotic kinases at four specific sites during mitosis, a time when nucleoplasm and cytoplasm mix. To understand the functional consequences of phosphorylation, we generated a panel of stable cell lines inducibly over-expressing PABPN1 with point mutations at these sites. Phospho-inhibitory mutations decreased cell proliferation, highlighting the importance of PABPN1 phosphorylation in cycling cells. Dynamic regulation of poly(A) tail length and RNA stability have emerged as important modes of gene regulation. We therefore employed long-read sequencing to determine how PABPN1 phospho-site mutants affected poly(A) tails lengths and TimeLapse-seq to monitor mRNA synthesis and decay. Widespread poly(A) tail lengthening was observed for phospho-inhibitory PABPN1 mutants. In contrast, expression of phospho-mimetic PABPN1 resulted in shorter poly(A) tails with increased non-A nucleotides, in addition to increased transcription and reduced stability of a distinct cohort of mRNAs. Taken together, PABPN1 phosphorylation remodels poly(A) tails and increases mRNA turnover, supporting the model that enhanced transcriptome dynamics reset gene expression programs across the cell cycle.

## Graphical Abstract

**Graphical Abstract:**
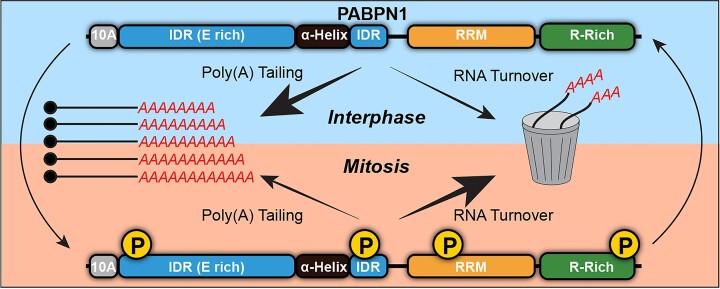

## Introduction

Pre-mRNA 3′-end processing constitutes a critical step in shaping gene expression programs in human cells (1). 3′-End processing requires dozens of proteins that select and cleave RNAs at the correct sequence and then coordinate processive poly(A) tail synthesis (2). Poly(A) tail synthesis is the final step of pre-mRNA processing prior to mRNA export to the cytoplasm where the poly(A) tail is required for efficient translation and protection from endonucleases (2–4). Poly(A) tail lengths are dynamic throughout the lifetime of an mRNA and provide a post-transcriptional mechanism to tune gene expression (2). Notably, poly(A) tail shortening or extension in the cytoplasm are common mechanisms employed by several developmental programs, emphasizing the importance of precise control of poly(A) tail lengths for these functions (5,6).

In the nucleus, synthesis of poly(A) tails is carried out by poly(A) polymerase (PAP) immediately after 3′-end cleavage (1). However, PAP requires several auxiliary factors that enable it to synthesize full-length poly(A) tails (∼250 nucleotides in mammalian cells). One such factor is the nuclear poly(A) binding protein (PABPN1), which oligomerizes on the growing tail and stabilizes PAP until the mature poly(A) tail is synthesized (7,8). Aberrant oligomerization caused by an increase in poly-alanine in the N-terminal ‘10A’ domain leads to Oculopharyngeal Muscular Dystrophy (9,10). Though the primary activity of PABPN1 is to promote polyadenylation, several roles for PABPN1 have emerged since its initial discovery. Notably, PABPN1 can target nuclear poly(A)+ RNA for degradation through direct interactions with the Poly(A) tail Exosome Targeting (PAXT) complex, and it has also been implicated in regulated alternative 3′-end cleavage, splicing, and nuclear export of mRNAs (11–15). Consistent with the coordination between PABPN1 and other pre-mRNA processing factors, PABPN1 is largely restricted to the nucleus where it is enriched in nuclear speckles, membraneless organelles that house many pre-mRNA processing factors (16,17).

Mitosis is a unique stage of the cell cycle that poses specific challenges with respect to pre-mRNA synthesis and processing. During mitosis, the nuclear envelope is broken down, the nuclear and cytoplasmic compartments mix, and membraneless organelles including nuclear speckles are disassembled (18). Transcription is also globally shut off during mitosis (19), yet poly(A) tail lengths remain largely unchanged despite the abundance of mRNA targets and the lack of new pre-mRNAs acting as a sink (20). PABPN1 was previously shown to be capable of recruiting and stabilizing PAP on RNAs with mature poly(A) tails independent of pre-mRNA cleavage (21). Therefore, mitosis may represent a timepoint when the mature transcriptome is vulnerable to re-binding by PABPN1 and aberrant poly(A) tail extension and/or targeting for decay. Moreover, a recent study identified waves of mRNA decay that occur during the mitosis-to-G1 phase transition (22), showing the potential relevance of PABP-directed decay mechanisms to cell cycle. All of these observations beg the question of how RNA processing factors are regulated during cell cycle to ensure early gene expression control in the resulting daughter cells.

In this study, we discovered that PABPN1 is phosphorylated in mitotic cells. After identifying four previously unknown phosphorylation sites and providing evidence that mitotic kinases are responsible for installing these changes, we sought to create an experimental platform to understand the impact of PABPN1 phosphorylation on precursor and mature mRNA processing. To do so, we established a series of stable human cell lines over-expressing WT, phospho-mimetic and phospho-inhibitory forms of PABPN1 enabling us to query the effects of mutations on steady-state transcriptomes, RNA turnover rates, poly(A) tail lengths, and cell proliferation itself. This comprehensive approach reveals important role(s) for PABPN1 regulation in transcriptome dynamics and cell cycle, including the requirement for PABPN1 phosphorylation for cell proliferation. In particular, our findings demonstrate a major mechanistic link between PABPN1 activity and mRNA stability control as cells passage through the cell cycle.

## Materials and methods

### Cell culture

HeLa cells (AVAM672) and HCT116 cells were cultivated in Dulbecco's modified Eagle's medium GLutaMAX (DMEM) (Gibco) supplemented with 10% fetal bovine serum (Gibco) and 1% penicillin/streptomycin (Gibco). HEK293 PABPN1 mutant cell lines were cultivated at 37°C in DMEM supplemented with 10% tetracycline-free FBS (Gibco), 1% penicillin/streptomycin and 10 μg/ml blasticidin (Gibco).

### PABPN1 mutant cell line preparation

Cell lines capable of inducible overexpression of PABPN1 WT or phospho-mutants were prepared by transfection (FuGene HD, Promega) of HEK293 FLP-In T-Rex cells with two plasmids: pOG44 to express the FLP recombinase and a second plasmid containing the respective PABPN1 WT or mutant sequence (all derived from pcDNA5/FRT/TO, Invitrogen). Empty vector cell lines had the unmodified pcDNA5/FRT/TO as the second plasmid. After the transfection, cells were selected for with 200 μg/ml Hygromycin and 15 μg/ml Blasticidin for 3 weeks with continuous media changes every 2–3 days. All antibiotic-resistant cells were taken from a plate (containing >100 colonies). PABPN1-mCherry expressing cells were prepared by lentiviral transduction of a plasmid encoding PABPN1-mCherry (pDVP26) into target HeLa AVAM672 cells. mCherry-expressing cells were then selected for using fluorescence-activated cell sorting (FACS Aria III sorter).

### Mitotic arrest and cell cycle synchronization

**Figure 1.**
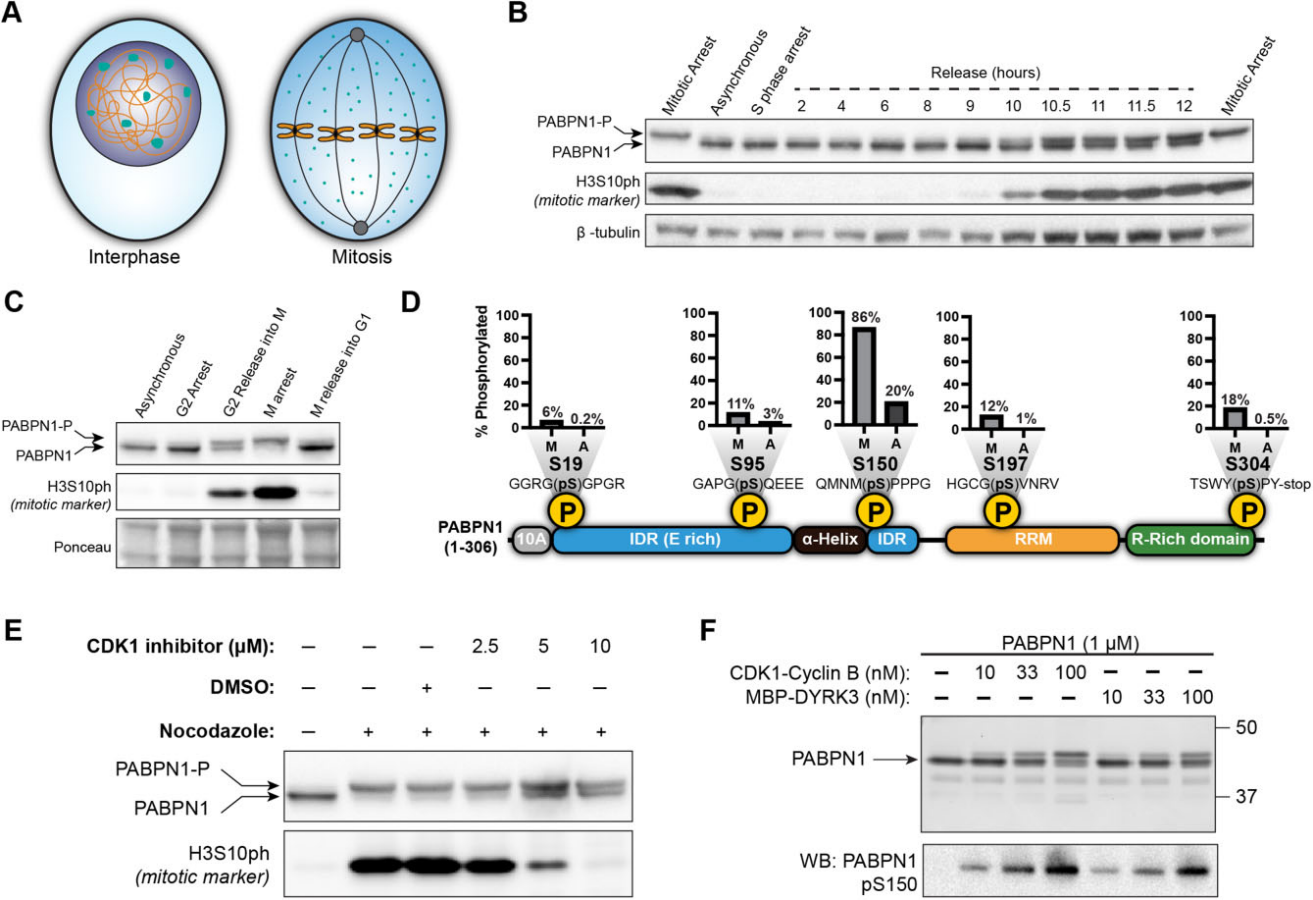
PABPN1 is phosphorylated during mitosis. (**A**) Schematic of interphase and mitotic cells. Nuclear speckles are depicted in teal. (**B**) Anti-PABPN1 and anti-H3S10ph western blots of extracts of HeLa cells arrested in S-phase or released into mitosis. Anti-β-tubulin was used to assess loading. (**C**) Anti-PABPN1 and anti-H3S10ph western blots of extracts of HeLa cells at either asynchronous, G2 arrested, G2 released, mitotic arrested, or mitotic released time points. Ponceau stain was used to assess loading. (**D**) Percent phosphorylation for PABPN1-mapped peptides isolated from mitotic and asynchronous HeLa cells determined by mass spectrometry. Position of phosphorylation site on PABPN1 domain architecture is indicated. Amino acid sequences surrounding phosphorylation sites are also displayed. (**E**) Anti-PABPN1 and anti-H3S10ph western blots of extracts from HeLa cells treated with CDK1 inhibitor RO-3306 in the context of mitotic arrest with nocodazole. (**F**) Coomassie stain and anti-pS150 PABPN1 western blots of recombinant PABPN1 incubated with recombinant CDK1-Cyclin B or MBP-DYRK3-His.

For cell cycle synchronization in Figure 1B and Supplementary Figure S1A, cells were synchronized in early S phase with a double thymidine block (DTB) and subsequently released, and then samples were collected for analysis at the indicated time points. To this end, cells in 10 cm plates were arrested in a first thymidine block by adding 2.5 mM thymidine for 18 h, then released by washing multiples times with fresh media. A second thymidine block was added 10 h after release and remained in place for ∼14 h, and cells were again released by washing multiple times with fresh media. Cells were harvested by gently scraping off the plate, washing with PBS, transferring to 1.5 ml tubes, removing the PBS and snap-freezing in liquid nitrogen. Asynchronous cells were harvested from an untreated plate. The mitotic arrested sample was treated with 100 ng/ml nocodazole 6 h after DTB release, cultured for another 7 h, and then collected by mitotic shake-off. For mitotic kinase-inhibitor experiments in Supplementary Figures S1C, samples were collected by mitotic shake-off and then treated with DMSO versus the indicated kinase inhibitors for 1 h.

In Figures 1C and E, cells were released from a single thymidine block into either a CDK1 inhibitor for a G2 arrest (10 μm RO-3306) or 100 ng/ml nocodazole for a mitotic arrest. In Figure 1E, the mitotic-arrested cells in were subsequently treated with 2.5–10 μm RO-3306 for 45 min to test the CDK1-dependence of PABPN1 phosphorylation. In Figure 1C, the G2-arrested and mitotic-arrested cells were washed and allowed to progress in the cell cycle for the indicated times before being harvested.

### Western blotting

Western blots were carried out as previously described using antibodies specific for the following proteins/epitopes: GAPDH (abcam ab9485), PABPN1 (abcam ab75855), LAMIN B1 (abcam ab16048), β-tubulin (abcam ab179511), H3S10ph (abcam ab308373), pS150 PABPN1 (Invitrogen, PA5-105091). Total RNA was stained with Ponceau S (Thermo Scientific) as recommended by the manufacturer. Western blots were developed with ECL Western Blotting Detection Kit (Cytiva Amersham) and visualized using a Bio-Rad GelDoc imaging system.

### Immunostaining and confocal microscopy

Cells were grown on No 1.5 coverslips (Zeiss) or 6 well plates for 24 h. Cells were then fixed in 4% paraformaldehyde, permeabilized in 0.25% Triton-X100 (American Bioanalytical) in PBS, blocked in 3% Bovine Serum Albumin (Sigma), and probed with the following antibodies in blocking buffer: Primary antibodies: mouse anti-SC35 and rabbit anti-PABPN1; Secondary antibodies: anti-rabbit Alexa-594 and anti-mouse Alexa-488 (Jackson laboratories). Cell nuclei were stained with 0.25 μg/ml Hoechst 34 580 (Thermo Fisher Scientific) in PBS and mounted using Prolong Diamond Antifade Mountant (Thermo Fisher Scientific). Imaging was carried out using a Leica SP8 Laser Scanning Confocal. Maximum intensity projections were generated from images using Fiji (23). Using the Hoechst signal, nuclei were segmented using the Cellpose neural network (24). For individual nuclei, Pearson's correlation of pixel values was calculated between the PABPN1 and SC35 channels. Statistical testing between conditions was performed using a Mann–Whitney *U* test.

### Immunoprecipitation

HeLa cells expressing PABPN1-mCherry were harvested by scraping in ice cold PBS followed by centrifugation at 270 × g for 10 min. Cell pellets were washed twice in ice cold PBS and incubated with lysis buffer (50 mM HEPES pH 7.4, 150 mM KCl, 2 mM EDTA, 0.1% NP-40, 5% glycerol, cOmplete protease inhibitor (2× recommended concentration, Roche), PhosSTOP inhibitor (2× recommended concentration, Roche), 1 mM PMSF, 10 mM β-glycerophosphate; 1.2 ml for 2 million cells). Cells were then sonicated for 90 s at 15% amplitude on ice (10 s On, 10 s Off), alternating every 30 s between samples. Samples were centrifuged at 10 000 × g, 4°C 10 min to clear lysate. 6 μg of anti-mCherry (Rat, Invitrogen, M11217) per IP were added per sample and incubated at 4°C O/N. SureBeads (Protein G, BioRad) or Dynabeads (Protein G, Invitrogen) were used according to the manufacturer to pull down anti-mCherry. Anti-PABPN1 IP’s from cells overexpressing EV, WT, 2SA, 4SA, 2SD or 4SD PABPN1 (following 3 day induction with 1 μg/ml DOX) were carried out as described above with the following modifications: 2.5 μg of anti-PABPN1 (abcam, ab75855) were used. For RNase-treated samples, 25 μg of RNase A were added prior to pulldown and incubated at 25°C for 20 min.

### Protein purifications

His-MBP-PABPN1 was expressed in BL21-CodonPlus (DE3)-RIPL cells (Agilent). Cells were grown O/N at 16°C following induction by 1 mM IPTG (Invitrogen). Cells were harvested by centrifugation (5000 × g, 15 min, 4°C) and resuspended in lysis buffer (60 mM Tris pH 8.0, 250 mM KCl, 5 mM MgCl_2_, 5% glycerol, 0.05% NP-40, 0.25 mM DTT, 1 mM PMSF, supplemented with cOmplete protease inhibitor EDTA-free; 1 ml buffer for every 10 ml of culture). Lysozyme (Sigma) was added to a final concentration of 100 μg/ml and samples were incubated with rotation for 30 min at 4°C. Samples were then sonicated for 2 min (5 s on, 5 s off) on ice. Lysate was cleared by centrifugation at 15 000 rpm for 30 min. 5 ml of Ni-NTA resin (HisPur, Thermo Scientific) was equilibrated with wash buffer (50 mM Tris pH 8.0, 300 mM KCl, 5 mM MgCl_2_, 5% glycerol, 0.02% NP-40, 1 mM PMSF, supplemented with cOmplete protease inhibitor EDTA-free). Imidazole (Thermo Scientific) was added to 15 mM to cleared lysate and incubated with Ni-NTA resin at 4°C O/N. Resin was poured over a filtered glass gravity column and flowthrough collected. The resin was then washed with 40 ml of ice-cold wash buffer, followed by five washes with 5 ml of wash buffer containing 350 mM imidazole while collecting fractions). His-MBP-PABPN1 content for each fraction was assessed by SDS-PAGE and His-MBP-PABPN1-containing fractions were pooled and dialyzed into wash buffer O/N. His-MBP tag was cleaved by O/N treatment at 4°C with TEV protease (∼1.7 mg, New England Biolabs). Cleaved His-MBP and uncleaved protein were removed using Ni-NTA resin as described above. The resulting PABPN1 was dialyzed into a buffer containing 25 mM Tris pH 8.0, 5 mM MgCl_2_, 5% glycerol, 0.02% NP-40, 100 mM KCl, 1 mM ATP and further purified using a HiTrap Q HP Anion Exchange column (Cytiva). The PABPN1-containing sample was concentrated to ∼2 ml using Amicon Ultra Centrifugal Filter tubes (10 kDa MW cutoff) and loaded onto the column. PABPN1 was eluted off the column with a linear gradient from 100 mM KCl to 1 M KCl (10 1 ml fractions). Fractions were assessed for PABPN1 content by SDS-PAGE and PABPN1-containing fractions were pooled, buffer-exchanged (Amicon) into storage buffer (50 mM Tris–HCl, pH 8.0, 10% glycerol, 1 mM EDTA, 100 mM KCl) and stored at −80°C. His-MBP-GFP was prepared essentially as described for His-MBP-PABPN1.

For RNA binding assays, expression vectors for 4SA and 4SD PABPN1 were generated using the QuickChange Lightning mutagenesis kit (Agilent). WT, 4SA, and 4SD His-MBP-PABPN1 were expressed and purified as follows. His-MBP-PABPN1 constructs were expressed in BL21-CodonPlus (DE3)-RIPL cells and lysate was prepared as described above. For consistency between WT, 4SA, and 4SD His-MBP-PABPN1, proteins were purified using an ÄKTA pure fast protein liquid chromatography system (Cytiva). First, clarified lysate was loaded onto a pre-equilibrated 5 ml HisTrap HP column (Cytiva). The column was washed with 6 column volumes of Buffer A containing 50 mM Tris pH 8.0, 300 mM KCl, 5 mM MgCl_2_, 5% glycerol, 0.02% NP-40, 1 mM PMSF, 15 mM Imidazole, supplemented with cOmplete protease inhibitor EDTA-free. The column was then washed with 10 column volumes of 90% Buffer A, 10% Buffer B containing 50 mM Tris pH 8.0, 300 mM KCl, 5 mM MgCl_2_, 5% Glycerol, 0.02% NP-40, 1 mM PMSF, 500 mM imidazole. Protein was then eluted in 5 column volumes of Buffer B while collecting 4 ml fractions. All HisTrap purification steps were performed at 4°C with a flow rate of 1 ml/min. His-MBP-PABPN1 content for each fraction was assessed by SDS-PAGE and His-MBP-PABPN1-containing fractions were pooled and dialyzed into buffer containing 25 mM Tris pH 8.0, 5 mM MgCl_2_, 5% glycerol, 0.02% NP-40, 100 mM KCl. Samples were concentrated to 5 ml and further purified using a Mono Q 10/100 GL column (Cytiva). Samples were loaded onto a pre-equilibrated Mono Q column. The column was then washed with 5 column volumes of Buffer A containing 25 mM Tris pH 8.0, 5 mM MgCl_2_, 5% glycerol, 0.02% NP-40, 100 mM KCl. Protein was eluted over a 15-column volume gradient from 20% Buffer B (25 mM Tris pH 8.0, 5 mM MgCl_2_, 5% glycerol, 0.02% NP-40, 1 M KCl) to 100% Buffer B, followed by 5 column volumes of 100% Buffer B, collecting 4 ml fractions. All Mono Q purification steps were performed at 4°C with a flow rate of 4 ml/min. His-MBP-PABPN1 content for each fraction was assessed by SDS-PAGE and His-MBP-PABPN1-containing fractions were pooled and concentrated to 2 ml. Samples were then aliquoted and stored at −80°C.

MBP-DYRK3-His was expressed in BL21 (DE3) cells (Agilent) and grown for 5 h at 22°C following induction by 1 mM IPTG. Cells were harvested by centrifugation at 5000 × g and resuspended in lysis buffer (25 mM HEPES pH 7.4, 5 mM MgCl_2_, 0.02% NP-40, 1 mM DTT, 2 mM ATP, 15 mM imidazole). Cells were lysed and lysate cleared as described above. 5 ml of Ni-NTA resin was equilibrated with lysis buffer supplemented with 300 mM KCl. Lysate was incubated with Ni-NTA resin for 30 min, 4°C and poured over glass gravity column. Resin was then washed with 30 column volumes of lysis buffer supplemented with 400 mM KCl. MBP-DYRK3-His was eluted with 4 column volumes of lysis buffer supplemented with 300 mM KCl, 350 mM Imidazole (5 ml fractions collected). MBP-DYRK3-His was assessed by SDS-PAGE and MBP-DYRK3-His-containing fractions pooled. MBP-DYRK3-His was further purified by amylose affinity purification. 1 ml amylose resin (NEB) was equilibrated with lysis buffer and incubated with Ni-NTA eluates for 20 min at 4°C. The resin was loaded onto a glass gravity flow column and washed with 20 ml of lysis buffer supplemented with 300 mM KCl, 20 ml of lysis buffer supplemented with 500 mM KCl, 15 ml of lysis buffer supplemented with 150 mM potassium acetate, and finally 15 ml of lysis buffer supplemented with 300 mM potassium acetate and 10% glycerol. MBP-DYRK3-His was eluted from the resin with lysis buffer supplemented with 5 column volumes of lysis buffer supplemented with 300 mM potassium acetate, 10% glycerol and 10 mM maltose. MBP-DYRK3-His content was assessed by SDS-PAGE and MBP-DYRK3-His-containing fractions were pooled and concentrated.

### Filter binding assays

Filter binding assays were performed as previously described (25). ^32^P-labeled poly(A)_30_ RNA was prepared by enzymatically end labelling an RNA oligo consisting of 30 consecutive A’s (Integrated DNA Technologies) with γ-^32^P ATP (Revvity) using T4 Polynucleotide Kinase (New England Biolabs) as recommended by the manufacturer. Unlabeled nucleotides were removed with microspin G-25 columns (Cytiva). His-MBP-PABPN1 (WT, 4SA or 4SD) or GFP were serial diluted from 4 μM to 62.5 nM in binding buffer containing 50 mM Tris–HCl pH 8.0, 10% glycerol, 0.2 mg/ml BSA, 0.01% NP-40 0.5 mM DTT, 100 mM KCl, 2 mM EDTA, 2 U/μl RNase OUT (Thermo), 5 mM MgCl_2_. Serial diluted PABPN1 in 10 μl reactions was incubated with 500 pM ^32^P-labeled poly(A)_30_ for 30 min at room temperature. Reactions were loaded onto a pre-wet sandwich of HyBond nylon membrane (Amersham) and nitrocellulose filter membrane (Bio-Rad) sealed between a Bio-Dot SF microfiltration apparatus (Bio-Rad). Samples were gently pulled through the membranes and wells were washed with 100 μl of binding buffer. Both membranes were then removed from the apparatus and allowed to dry. Dried membranes were exposed to a storage phosphor screen and the screen was imaged on a phosphor imager (Bio-Rad Personal Molecular Imager system). Assays were performed in biological triplicate (PABPN1 constructs) or duplicate (GFP control). Signal intensity for each dot was quantified using Fiji (23). Percent RNA bound for each reaction was calculated as: [intensity nitrocellulose] / ([intensity nitrocellulose] + [intensity nylon]). The resulting binding curves were fit to the equation:


\begin{equation*}{Fraction}_{bound} = {Fraction}_{maxbound}[{PABPN1}]/([{PABPN1}] + {K}_{d})\end{equation*}

### Sample preparation for mass spectrometry of PABPN1 in asynchronous and mitotic cells

PABPN1-mCherry expressing HeLa cells were grown to roughly 60% confluency in 15 cm plates. To arrest cells in mitosis, cells were treated with 100 ng/μl of nocodazole for 14 h. Mitotic cells were collected by mitotic shake-off and collecting unattached cells. Cells were washed with PBS and collected. Interphase cells (untreated) were collected by mitotic shake-off and removing media, washing cells with PBS (wash discarded), and adherent cells scraped off the plate. Cells were harvested by centrifugation as described above and lysed in lysis buffer (50 mM HEPES pH 7.4, 150 mM KCl, 2 mM EDTA, 1% NP-40, 5% glycerol, cOmplete protease inhibitor (Roche), PhosSTOP inhibitors (Roche), 1mM PMSF, 10 mM β-glycerophosphate). PABPN1-mCherry was immunoprecipitated as described above. IP samples were run on an SDS-PAGE gel and PABPN1-mCherry was cut from the gel. Excised gel bands were submitted to the Keck MS & Proteomics Resource at the Yale School of Medicine for mass spectrometry analyses. Sample preparation of the gel bands was performed according to a previously published method with slight modification. Briefly, excised gel bands were initially washed with 1 ml of water for 10 min; then followed by a 20 min wash with 1 ml of a 50% acetonitrile (ACN): 50% 100 mM ammonium bicarbonate (ABC) buffer. The proteins in the gel bands were then reduced with 125 μl of 4.5 mM dithiothreitol (DTT) at 37°C for 20 min and cooled to RT. Alkylation was carried out with 125 μl of 10 mM iodoacetamide at RT for 20 min in the dark. The gel bands were then washed with 1ml of a 50%ACN:50%H_2_O containing 100 mM ABC for 20 min followed by a final wash with 1 ml of a 50%ACN:50%H_2_O containing 25mM ABC for 20 min. The gel bands were then dried in a speed vac for 10 min. Trypsin digestion was then carried out (1:200 molar ratio of trypsin to protein) by incubation at 37°C O/N. Digest samples were then extracted with 300 μl of an 80%ACN:20%H_2_O containing 0.1% trifluoroacetic acid for 15 min. Extracted peptides were dried in a speed vac and reconstituted in UPLC loading Buffer A (100% water, 0.1% formic acid).

### Sample preparation for mass spectrometry of in vitro phosphorylation of PABPN1

Purified PABPN1 (1 μg) was incubated with either 0.27 μg of human CDK1-Cyclin B (ThermoFisher) or 0.32 μg of MBP-DYRK3-His in kinase buffer (50 mM Tris pH 8.0, 150 mM KCl, 5 mM MgCl_2_, 1 mM DTT, 0.02% NP-40, 200 μM ATP). Kinase reactions were incubated at 25°C for 30 min. Samples were run on an SDS-PAGE gel and phosphorylated and unphosphorylated PABPN1 bands were cut from the gel. Excised gel bands were submitted to the Keck MS & Proteomics Resource at the Yale School of Medicine for mass spectrometry analyses as described above.

### Sample preparation for mass spectrometry for PABPN1 interactome

PABPN1 phospho-mutants (WT, 4SA, 4SD) were seeded in 15 cm plates and mutant expression induced with 1 μg/ml DOX for 3 days. Cells were harvested by scraping or rinsing in ice cold PBS followed by centrifugation at 270 × g for 10 min. Cell pellets were washed twice in ice cold PBS. Cells were resuspended in 1 ml of lysis buffer (50 mM HEPES pH 7.4, 150 mM KCl, 2 mM EDTA, 5 mM MgCl_2_, 0.1% NP-40, cOmplete protease inhibitor (Roche), PhosSTOP inhibitor (Roche), 0.5 mM PMSF). Cells were lysed by sonication for 75 s at 15% amplitude on ice (15 s on, 15 s off). Lysate was cleared twice by centrifugation at 20 000 × g at 4°C for 10 min. PBS and RNase A-treated samples were prepared and anti-PABPN1 immunoprecipitations performed as described above. Samples were submitted to the Keck MS & Proteomics Resource at the Yale School of Medicine for mass spectrometry analyses as described above.

### Mass spectrometry

Peptides were analyzed by LC–MS/MS using a Q-Exactive Plus mass spectrometer equipped with a Waters nanoACQUITY ultra-performance liquid chromatography (UPLC) system or using a Waters Symmetry C18 180 mm by 20 mm trap column and a 1.7 mm (75 mm inner diameter by 250 mm) nanoACQUITY UPLC column (35°C) for peptide separation. Trapping was carried out at 5 μl/min, 99% Buffer A (100% water, 0.1% formic acid) for 3 min. Peptide separation was performed at 300 nl/min with a linear gradient that will reach 5% Buffer B (100% CH_3_CN, 0.075% formic acid) at 1 min, 25% B at 45 min and 50% B at 65 min, and 90% B at 70 min for 5 min; then dropdown to 3% Buffer B at 77 min for 5 min. For the LCMS/MS data dependent acquisition on the Q-Exactive Plus mass spectrometer, High-energy Collisional Dissociation (HCD) MS/MS spectra were filtered by dynamic exclusion (20 s) and acquired for the Top 20 peaks with charge states 2–6 with *m*/*z* isolation window of 1.7. All MS (Profile) and MS/MS (centroid) peaks were detected in the Orbitrap.

### Mass spectrometry data analysis

For the protein and protein post-translational modification experiments, mass spectral data were processed using Proteome Discoverer (PD; ThermoFisher Scientific, Waltham, MA; v. 2.3). Protein searches were conducted against the Homo sapiens SWISSProt protein database using Mascot Search Engine (Matrix Science, LLC, Boston, MA; v. 2.6.0). Mascot search parameters included: parent peptide ion tolerance of 10.0 ppm, peptide fragment ion mass tolerance of 0.020 Da, strict trypsin fragments (enzyme cleavage after the C terminus of K or R, but not if it is followed by P), variable modification by phosphorylation (S, T, Y, and H), oxidation (M), and propioamidation (C). PD results were then imported into Scaffold Q + S and Scaffold PTM software (Proteome Software, Portland, OR; v.4.3.2 and v.3.0, respectively) for visualization of the results and manual examination of the MS/MS spectra and the corresponding assigned fragment ions were conducted to verify the identified phosphopeptides. For Label Free Quantitative experiment, mass spectral data were processed using Progenesis QI (Waters Inc., Milford, MA; v.4.2). Analysis method is described elsewhere by Torregrossa *et al.* (26). Differential protein interaction was analyzed using Scaffold 3. WT and 4SA/4SD were directly compared using two-tailed *t*-test on iBAQ values. 4SA and 4SD were directly compared using two-tailed t-test on iBAQ values.

### Cell viability assay

Crystal violet viability assays were performed as previously described (27). 300 EV, WT, 2SA, 4SA, 2SD or 4SD HEK293 cells were seeded per well in 6-well tissue culture plates and mutant PABPN1 expression was induced with 1 μg/ml DOX 24 h after seeding. Cells were allowed to grow continuously with media changes every 2–3 days. After 2 weeks of growth, cells were washed with ice cold PBS and stained with 0.5% crystal violet dye in 25% methanol for 30 min. Stained cells were washed extensively with PBS and dried O/N at room temperature (RT). Plate images were scanned on a Bio-Rad GelDoc imaging system. Crystal violet dye was solubilized in 20% acetic acid (4 ml per well) and absorbance at 590 nm was quantified on a plate reader (Molecular Devices Spectra Max m5). A^590^ for DOX treated cells/PBS treated cells for respective pairs was calculated in Microsoft Excel and two-tailed t-tests were used to determine statistical significance.

### Immunofluorescence staining for flow cytometry cell cycle analysis

Overexpression of EV, WT, 2SA, 4SA, 2SD or 4SD PABPN1 was induced by treatment with 1 μg/ml Doxycycline (DOX) (Millipore Sigma) for 3 days. Cells were harvested by rinsing off of the plate in growth media to avoid loss of non-adherent mitotic cells, followed by centrifugation at 270 × g for 10 min. Cell pellets were washed twice with 5 ml of ice-cold PBS, resuspended in 200 μl of PBS, and fixed in ice-cold 70% ethanol overnight at −20°C. 1 000 000 cells were aliquoted into new 15 ml Falcon tubes (Corning) and washed three times by centrifugation at 500 × g for 10 min and resuspension in 1 ml ice cold of 0.5% Tween 20, 1% BSA in PBS before staining overnight at 4°C with 2.5 μg of anti-H3S10ph Alexa Fluor 488 (abcam, ab197502) in 1 ml of ice cold 0.5% Tween 20, 1% BSA in PBS, protected from light. Cells were then washed twice by centrifugation at 500 × g for 10 min and resuspension in 1 ml ice cold of PBS before resuspending cell pellets in 1 ml of ice-cold PBS. Cells were stained with DNA dye FxCycle Violet (Invitrogen) according to the manufacturer. Samples were filtered into 5 ml round bottom polystyrene tubes (Corning) immediately before flow cytometry analysis.

### Flow cytometry analysis

Flow cytometry was performed on a BD LSR Fortessa using DIVA software (BD Biosciences). DNA content was measured by 405 nm excitation and 450/50 bandpass emission filter. Anti-H3S10ph Alexa 488 was measured by 488 nm excitation and 515/20 bandpass emission filter. Doublet discrimination was carried out using FxCycle Violet A and FxCycle Violet H measurements and data collection was halted at 30 000 G1 events. Data analysis was performed with Flowjo software. Cell cycle distribution was plotted as FxCycle Violet A versus log_10_ (Anti-H3S10ph Alexa 488).

### Northern blotting

Overexpression of EV, WT, 2SA, 4SA, 2SD or 4SD PABPN1 was induced by treatment with 1 μg/ml Doxycycline (DOX) (Millipore Sigma) for 3 days. Cells were harvested by scraping or rinsing in ice cold PBS followed by centrifugation at 270 × g for 10 min. Cell pellets were washed twice in ice cold PBS and either used immediately or flash frozen in liquid nitrogen and stored at −80°C. Northern blots were prepared as previously described (21). Briefly, total RNA was isolated from cells using TRIzol (Thermo Scientific) according to the manufacturer and digested with RNase T1 (Thermo Scientific). RNA samples were then mixed 1:1 with RNA loading dye (95% formamide, 0.025% SDS, 0.025% bromophenol blue, 0.025% xylene cyanol FF, 0.5 mM EDTA.), run on a 1.6% agarose formaldehyde gel, and capillary transferred overnight (O/N) to a Hybond-N+ membrane for northern probing (Cytiva Amersham). RNA was UV crosslinked to the membrane and the size markers (RiboRuler Low Range and RiboRuler High Range, Thermo Scientific) were visualized by staining with methylene blue. Membranes were prehybridized in ULTRAhyb buffer (Invitrogen) for 3 h at 42°C in a rotating incubator. Single-stranded DNA Oligo dT_40_ probes (Integrated DNA Technologies) were end labeled enzymatically with T4 Polynucleotide Kinase (New England Biolabs) using γ-^32^P ATP (Perkin Elmer) as recommended by the manufacturer. Membranes were incubated with the prepared probe in ULTRAhyb buffer O/N at 42°C and washed 3× for 30 min in 2× saline-sodium citrate buffer at 42°C. Blots were wrapped in plastic wrap and placed in an exposure cassette with radio-sensitive film for 2 weeks prior to development and imaging.

### PacBio sequencing library preparation

Overexpression of EV, WT, 4SA or 4SD PABPN1 was induced by treatment with 1 μg/ml DOX (Millipore Sigma) for 3 days. Cells were harvested by scraping or rinsing in ice cold PBS followed by centrifugation at 270 × g for 10 min. Cell pellets were washed twice in ice cold PBS. Two biological replicates were prepared for each cell line. Total RNA was isolated from cells using TRIzol as recommended by the manufacturer with the following modifications: After centrifugation of TRIzol/chloroform, the aqueous layer (250 μl) was removed, and RNA isolated using the Qiagen RNeasy kit. The resulting RNA was rRNA depleted using the RiboMinus cleanup kit (Invitrogen) and concentrated using an RNA clean and concentrate kit (Zymogen). The resulting RNA was then reverse transcribed with TGIRT-III reverse transcriptase (InGex) using a custom RNA/DNA duplex reverse primer (see Supplementary Table S1). Following reverse transcription, RNA was digested with RNase H (New England Biolabs) to remove any bound TGIRT-III and cDNA purified with the Qiagen MinElute kit. To prepare the 5′ adapter, single stranded DNA adapter oligo was treated with Mth ligase (New England Biolabs). The prepared adapter was then ligated to the cDNA pool using Thermostable 5′ App DNA/RNA Ligase (New England Biolabs) and again purified with the Qiagen MinElute kit. PCR amplification of cDNA library was carried out with Advantage PCR kit (Takara Bio) with primers that anneal to the 5′ and 3′ adapter sequences. Samples were barcoded with overhanging reverse primers and amplified by 18 cycles of PCR. DNA purification was carried out using AMPure beads (Beckman Coulter) and pooled at equimolar ratios. The PacBio SMRTbell prep kit was used for final library preparation according to the manufacturer (Pacific Biosciences) and sequenced on a PacBio Sequel II Long-Read Sequencer. Replicates 1 and 2 were sequenced on separate flow cells.

### Illumina sequencing library preparation

Overexpression of EV, WT, 4SA or 4SD PABPN1 was induced by treatment with 1 μg/ml DOX (Millipore Sigma) for 3 days. Cells were harvested by scraping or rinsing in ice cold PBS followed by centrifugation at 270 × g for 10 min. Cell pellets were washed twice in ice cold PBS. Three biological replicates were prepared for each cell line. Total RNA was isolated from cells using TRIzol and used to prepare RNA-seq libraries using the KAPA mRNA HyperPrep Kit (Kapa Biosystems). The RNA-seq library was sequenced on the Illumina Novaseq 6000 platform generating ∼30 million paired-end reads per sample (see Supplementary Table S2).

### TimeLapse-seq library preparation

Overexpression of EV, WT, 4SA or 4SD PABPN1 was induced by treatment with 1 μg/ml DOX (Millipore Sigma) for 3 days. TimeLapse-Seq was performed essentially as described in Schofield *et al.* (28). Briefly, during the last 2 h of DOX induction, 100 μM s^4^U was added to all samples (200 μM s^4^U for empty vector samples). Three biological replicates were prepared for each cell line. A single replicate of each cell line received DMSO (no s^4^U). Subsequent sample handling was performed in a manner to minimize light exposure. Cells were harvested by scraping or rinsing in ice cold PBS followed by centrifugation at 270 × g for 10 min. Cell pellets were washed twice in ice cold PBS. RNA was extracted and isolated using TRIzol as recommended by the manufacturer. RNA was precipitated in isopropanol supplemented with 1 mM DTT and 20 μg glycogen and washed with freshly prepared 75% ethanol. Dried pellets were resuspended in nuclease-free water. Turbo DNase was used to deplete any remaining genomic DNA. RNAClean beads (Beckman) were used to isolate the RNA, which was then subjected to TimeLapse chemistry (2,2,2-trifluoroethylamine and mCPBA) and further purified with RNAClean beads. This recoded RNA was prepared for sequencing using the mammalian pico-input SMARTer stranded Total RNA-Seq kit v2 (Takara Bio) according to the manufacturer. The RNA-seq library was sequenced on the Illumina Novaseq 6000 platform generating ∼100 nucleotide long paired-end reads.

### Long read sequencing data processing

HiFi circular consensus sequencing (CCS) reads were generated in .fastq format. Cutadapt (29) was used to demultiplex conditions based on barcode sequence and trim external adapter sequences with settings –cores = 14 –discard-untrimmed –no-indels –revcomp −e 0.2. Prinseq-lite (30) was used to remove PCR duplicates with settings −derep 2 −out_format 3. Prinseq-lite was used again with settings -trim_left 5 -out_format 3 to trim the UMI sequence. A genome for mapping was constructed by appending the sequence for the WT PABPN1 Flp-In plasmid to the GRCh38 (hg38) genome. Reads were mapped to the custom genome using minimap2 (31) with settings -x splice:hq –secondary no -t 12 -a -u f. Samtools (32) sort was used to sort reads by genomic position. Samtools view with settings -S -b was used to convert .sam files to .bam files. Samtools index was used to generate index files corresponding to each .bam file. Samtools view was also used to separate reads mapping to hg38 from those mapping to the plasmid sequence. Bedtools (33) bamtobed with setting -bed12 was used to generate bed12 files from .bam files. The flair software suite (34) was used to assign reads to splice isoforms, quantify isoform usage between samples, and mark isoforms with retained introns. Poly(A) tail lengths were counted using a custom script that counts soft-clipped bases on 3′-ends of reads (85% adenine requirement for tails). Poly(A) tail metrics per gene and per splice isoform were calculated by first overlapping shared regions between a gene annotation file (hg38 genes) and mapped reads. A custom script was used to calculate mean, median, pearson's-skew, and number of observations for each gene or splice isoform. For reproducibility, a Snakemake (35) pipeline that carries out the processing steps outlined above was generated. Non-A base usage in poly(A) tails was calculated using a custom script that aligns tails at either the poly(A) cleavage sites or 3′-ends and calculates the number of occurrences of each nucleotide at a given position. Plots were generated using matplotlib and seaborn python packages.

### Illumina sequencing data analysis

Paired-end read mates were generated in .fastq format. Illumina adapters were trimmed using fastp (36). Reads were mapped to a custom hg38/WT PABPN1 genome using STAR (37) with settings –runMode alignReads –outSAMtype BAM SortedByCoordinate –outFilterMismatchNmax 2 –alignIntronMin 20 –alignIntronMax 1 000 000 –alignMatesGapMax 1 000 000. Reads that mapped to the hg38 genome were separated from those mapping to exogenous sequence using samtools view with settings -F 4 and mapped files were indexed using samtools index. Read coverage was quantified using Salmon with settings −l A –validateMappings –gcBias. The QAPA software suite was used to analyze APA (38). For reproducibility, a Snakemake (35) pipeline that carries out the processing steps outlined above was generated. Quantified gene expression values were used to analyze differential gene expression with DESeq2 (39). Downstream comparison of gene expression between samples was carried out using a custom python script that utilizes the output files from DESeq2.

### TimeLapse-seq data processing

Paired-end read mates were generated in .fastq format. Illumina adapters were trimmed using Cutadapt (29). Reads were mapped to a custom hg38/WT PABPN1 genome using STAR (37) with settings –runMode alignReads –outSAMtype BAM SortedByCoordinate –outSAMattributes NH HI AS NM MD –quantMode TranscriptomeSAM GeneCounts –sjdbGTFfile [‘annotation’]. Mutation calling was also performed as previously described (28). Briefly, T-to-C mutations were not considered if the base quality score was <40. In addition, mutations within five nucleotides of the read's end were not considered. Sites of likely single-nucleotide polymorphisms (SNPs) and alignment artifacts (identified with bcftools v.1.11 (40)) were not considered in mutation calling. Multithread parallelization was implemented with GNU parallel 20210222. To facilitate reproducibility and external use of this pipeline, we used the Snakemake implementation of this workflow called bam2bakR, available on github (https://github.com/simonlabcode/bam2bakR) (41). The resulting files were used to carry out kinetic analyses using the bakR software suite (41). DESeq2 (39) was used to assess differential expression between conditions TimeLapse-seq data. Correlative analyses between synthesis and degradation kinetics and poly(A) tail lengths were carried out using custom python scripts.

## Results

### PABPN1 is phosphorylated in mitotic cells

RNA processing is regulated at various points in the cell cycle, accompanied by massive reorganization of the subcellular environment at mitosis (Figure 1A). To investigate the regulation of PABPN1 throughout the cell cycle, HeLa cells were synchronized in early S phase using double-thymidine block (DTB) and PABPN1 protein was analyzed (Figure 1B). Although PABPN1 protein levels were unchanged throughout the cell cycle, the protein exhibited an upward mobility shift 9 h after DTB release, suggestive of post-translational modification (PTM) coinciding with mitotic entry. We observed similar cell cycle regulation of PABPN1 in HCT116 cells (Supplementary Figure S1A). In both cell lines, the higher apparent molecular weight (MW) PABPN1 was most distinct when more cells were in mitosis, as assessed by the level of histone H3 phosphorylated on serine 10 (H3S10ph). To more precisely define the cell cycle timing of the PABPN1 mobility shift, we tested different arrest and release conditions relative to mitosis, G2 and G1 phases of the cell cycle (Figure 1C). In all cases, the PABPN1 shift was restricted to mitosis and not observed in G2 or G1 phase. To define the type of PTM on PABPN1, we used PABPN1-mCherry cell lines which also had a mitosis-specific mobility shift and showed that the mobility of immunoprecipitated PABPN1-mCherry could be reversed using lambda phosphatase (Supplementary Figure S1B). Thus, phosphorylation is responsible for the observed PABPN1 band shift.

To further identify the mitosis-specific PTMs on PABPN1, we immunoprecipitated PABPN1-mCherry from HeLa cells in interphase vs. mitotic cells. Using mass spectrometry, we identified five phosphorylation sites that were enriched in mitosis, including S19, S95, S150, S197 and S304 which span the entire domain architecture of PABPN1 (Figure 1D, Supplementary Table S3). Because S95 phosphorylation was relatively minor and was previously shown to be strongly induced by DNA damage agents (42), we focused on understanding the implications of PABPN1 phosphorylation on S19, S150, S197 and S304 in the context of mitosis. The eukaryotic cell cycle is in large part regulated by the activity of cyclin-dependent kinases, and CDK1-Cyclin B serves as an important regulator of cell division (43). CDK1-Cyclin B is also largely restricted to Ser/Thr residues with a proline at +1, making it a strong candidate to phosphorylate Ser150 and Ser304 (both of which have Pro at +1, Figure 1D) (44). Besides CDKs, DYRK and CLK kinases are also important regulators of the cell cycle and favor sites with nearby proline and arginine residues (44). DYRK3 has been implicated in the disassembly of nuclear speckles (where PABPN1 is concentrated) during mitosis (45), and CLK kinases are also capable of disassembling nuclear speckles when overexpressed (46). Therefore, PABPN1 may be a substrate of the mitotic kinases CDK1 and DYRK3.

To determine whether PABPN1 is phosphorylated by one or more of these mitotic-specific kinases, we treated HeLa cells with either the Pan-CDK inhibitor roscovitine or DYRK/CLK inhibitor cocktail (KH-CB19 and GSK626016) and evaluated the extent of PABPN1 phosphorylation. Pan-CDK inhibitors, but not DYRK/CLK inhibitors, were sufficient to reverse PABPN1 phosphorylation in mitotically arrested cells in both HeLa and HCT116 cells (Supplementary Figure S1C). Treatment with a CDK1-specific inhibitor also reversed PABPN1 phosphorylation in HeLa cells *in vivo* in a dose-dependent manner (Figure 1E). In biochemical experiments, purified CDK1-Cyclin B and recombinant DYRK3 were both able to phosphorylate purified PABPN1 as evidenced by mobility shift (Figure 1F), and specificity was confirmed using both mass spectrometry (see Supplementary Table S4) and immunoblotting with an antibody specific for PABPN1 phosphorylated on Serine 150 (pS150) (Figure 1F, Supplementary Figure S1D). Our results indicate that PABPN1 is specifically phosphorylated in mitotic cells mostly due to the activity of CDKs; however, the ability of DYRK3 to phosphorylate recombinant PABPN1 *in vitro* highlights the possibility that these sites could be targeted by kinases in other contexts.

### Phospho-inhibitory PABPN1 mutants exhibit reduced cell proliferation

**Figure 2.**
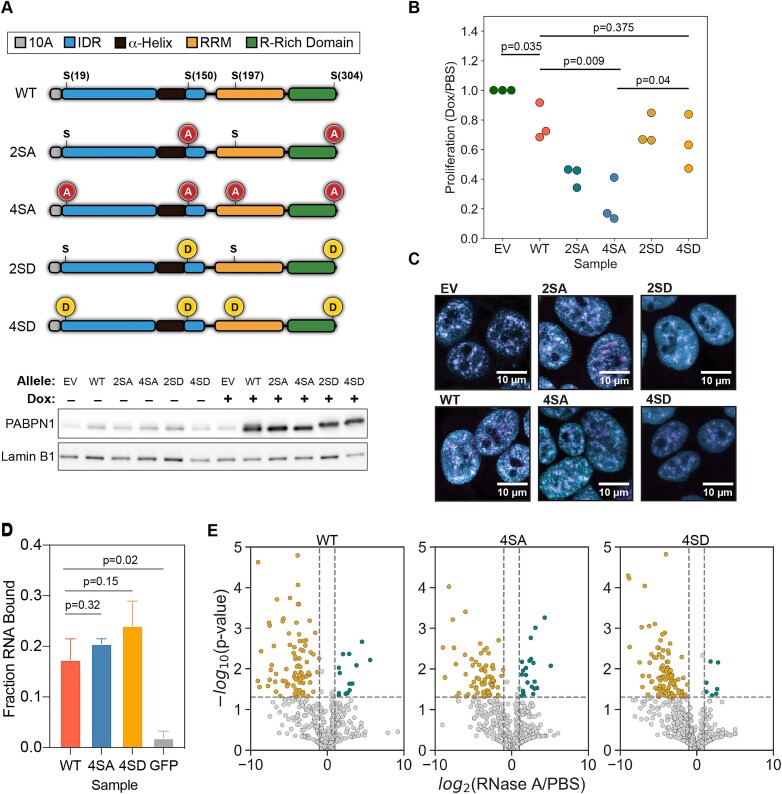
PABPN1 phospho-mutants have altered cell viability. (**A**) Schematic representing PABPN1 mutants that were generated to inhibit (Ser to Ala, red) or mimic (Ser to Asp, yellow) phosphorylation on mitosis specific residues (top). Positions of mutation sites are shown relative to the domain architecture of PABPN1. Anti-PABPN1 western blots of extracts of HEK293 cells following 72 h of DOX induction of PABPN1 mutant expression. Anti-Lamin B1 was used to assess loading (bottom). (**B**) Absorbance at 595 nm of crystal violet dye dissolved from each PABPN1 mutant sample. Values from DOX-treated samples were normalized to PBS-treated controls for each group and then to EV cells. Statistical significance was determined using two-tailed *t*-test. (**C**) Immunofluorescence microscopy images of PABPN1 phospho-mutant cell lines using anti-PABPN1 (cyan) and anti-SC35 (magenta) antibodies. Corresponding p-values for statistical testing are listed above pairs. (**D**) Fraction poly(A)_30_ bound (500 pM) after incubation with 1 μM PABPN1, measured by filter binding assay. Statistical significance was calculated using Welch's *t*-test. (**E**) Volcano plots of proteins that co-immunoprecipitated with WT (left), 4SA (middle) or 4SD (right) PABPN1, identified by mass spectrometry. Points in gold indicate enrichment in PBS treated (RNA-containing) samples. Points in teal indicate enrichment in RNase A-treated samples.

The mitotic phosphorylation sites S19, S150, S197 and S304 span several domains across PABPN1, with possible implications for multiple PABPN1 activities. To establish a robust system for analyzing the effects of PABPN1 mutations on cellular activities, including overall cell growth and proliferation, we therefore established a series of mutants that can be inducibly overexpressed in HEK293 cells (Figure 2A). The mutants included phospho-inhibitory alanine (S150A/S304A; or S19A/S150A/S197A/S304A, referred to as 2SA and 4SA respectively) or phospho-mimetic aspartate (S150D/S304D; or S19D/S150D/S197D/S304D, referred to as 2SD and 4SD respectively). Cells with empty vector (EV) or wild-type PABPN1 (WT) were also prepared as controls. All PABPN1 mutants were constructed using a HEK293 Flp-In parent cell line under the control of the cytomegalovirus promoter and tetracycline-response element for robust overexpression upon induction (Figure 2A, lower panel). All 5 cell lines (WT, 2SA, 4SA, 2SD and 4SD) expressed exogenous PABPN1 only in the presence of the tetracycline-analog DOX, and we also note that the 2SD and 4SD has a mobility shift similar to the phosphorylated PABPN1. EV and WT controls were also phosphorylated following mitotic arrest, while PABPN1 from 4SA and 4SD cells corresponded to constitutively unphosphorylated and phosphorylated respectively in both interphase and mitotic cells (Supplementary Figure S1E).

To test if PABPN1 phosphorylation affects cell proliferation, we used a crystal-violet viability assay. In brief, we grew our control and PABPN1 HEK293 cell lines under expression-inducing (DOX) or non-inducing (PBS) conditions for 14 days before staining with crystal-violet (Supplementary Figure S2A). For quantification, crystal violet dye from each sample was solubilized and quantified by absorbance at 595 nm (Figure 2B). Overexpression of WT PABPN1 resulted in a slight but significant defect in proliferation relative to the EV controls, in agreement with previous reports (47). A similar level of growth was also seen in the 2SD and 4SD mutants. However, the 2SA and 4SA mutants exhibited substantial proliferation defects compared to the other cell lines. Our results indicate that cells expressing phospho-inhibitory PABPN1 fail to proliferate as well as control samples or the phospho-mimetic mutants, indicating that the ability to phosphorylate PABPN1 is important for human cell growth or division.

To determine if PABPN1 phospho-mutant expression specifically alters cell cycle progression, we performed cell cycle analysis using flow cytometry (Supplementary Figure S2B). We grew our control and PABPN1 HEK293 cell lines under expression-inducing (DOX) conditions for 3 days, stained cells with a DNA dye and fluorescently tagged antibody against mitotic marker H3S10ph and analyzed the fluorescence intensity of each dye in single cells using flow cytometry. EV, WT, 4SA and 4SD cells all displayed similar fractions of cells in G1, S, G2 and mitosis, indicating that all four cell types are progressing through the cell cycle (Supplementary Figure S2C). Cells expressing 4SA PABPN1 did not accumulate in mitosis. However, a slight but significant decrease in the fraction of mitotic cells in 4SD compared to WT (Supplementary Figure S2D). Our results indicate that phospho-inhibitory PABPN1 expression does not lead to stalling in any cell cycle phase, suggesting that the cell cycle progresses at an overall slightly slower pace that is additive over multiple generations.

### PABPN1 phospho-mutants have similar localization and RNA-binding properties

We next investigated the subcellular localization of our PABPN1 phospho-mutants. Importantly, all five PABPN1 variants were exclusively nuclear at interphase (Figure 2C, Supplementary Figure S3A and S3B). Interestingly, we observed a slight but significant decrease in nuclear speckle overlap in 4SD compared to WT (Supplementary Figure S3C). However, the magnitude of this result does not indicate that PABPN1 localization is strongly affected by phosphorylation. Phosphorylation has been shown to more drastically regulate protein localization to membraneless organelles in many other contexts, indicating that these phosphorylation sites may regulate other aspects of PABPN1 function (48).

One possible effect of PABPN1 phosphorylation could be on its ability to bind RNA, especially since S197 occurs within the RRM (Figure 2A). To directly test this, we expressed and purified WT, 4SA and 4SD His-MBP-PABPN1 from *E. coli*. We then measured its ability to bind a 30-nucleotide long poly(A) oligo (poly(A)_30_) using a filter binding assay (49). All three proteins were capable of binding poly(A)_30_*in vitro* (Figure 2D, Supplementary Figure S4A), which may help explain why PABPN1’s localization to RNA-rich speckles is relatively unaffected.

The importance of PABPN1 phosphorylation for cell proliferation could also indicate a change in protein interaction partners. We analyzed protein interaction partners of PABPN1 using mass spectrometry after PABPN1 immunoprecipitation and carried out these assays in the presence or absence of RNase A to identify RNA independent and RNA dependent interactions. All forms of PABPN1 tested, including WT, 4SA and 4SD cells, yielded many protein interactors that were highly enriched in RNA binding proteins, most of which were RNA-dependent (Figure 2E, Supplementary Table S5). For example, hnRNP R was enriched in WT, 4SA and 4SD PABPN1 but was lost upon RNase treatment (Supplementary Figure S4B). While some overlap in RNA-dependent protein interactors were observed among WT, 4SA and 4SD PABPN1, there were numerous differences (Supplementary Figure S4C). The strong RNA dependence of these interactions is consistent with our finding that both 4SA and 4SD PABPN1 are still capable of binding RNA *in vitro*, indicating this is also true *in vivo*. Interestingly, 4SD PABPN1 yielded more RNA-dependent protein interactors than did 4SA PABPN1 (Supplementary Figure S4C and S4D). Because neither PAP nor components of PAXT were reproducibly detectable with PABPN1 by mass spectrometry (which recapitulates other reports (42)), we were unable to use this assay to predict whether polyadenylation or RNA decay were impacted by PABPN1 phosphorylation. Therefore, we designed several functional assays to test these possibilities.

### Expression of phospho-inhibitory PABPN1 results in RNA hyperadenylation

**Figure 3.**
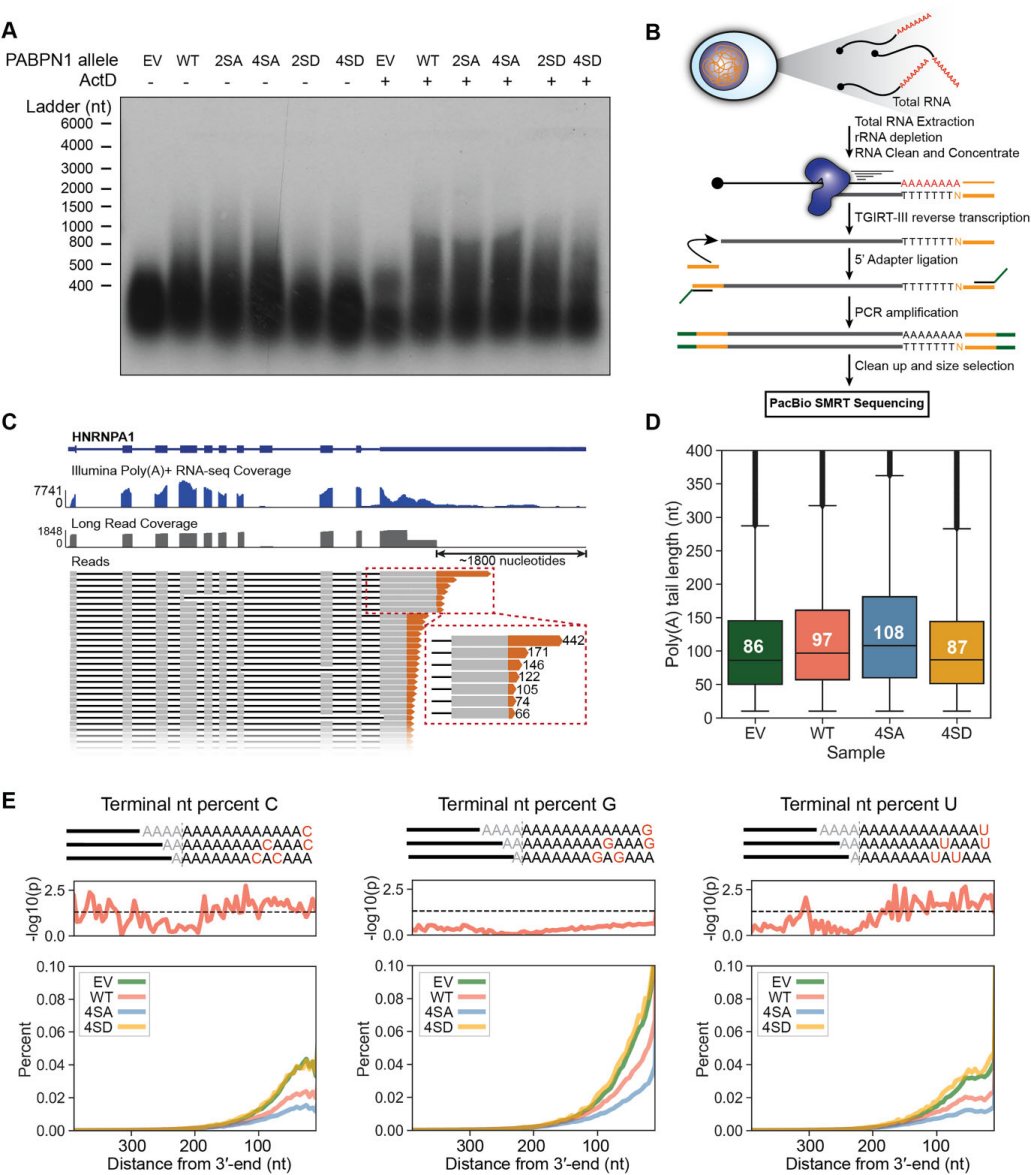
Analysis of poly(A) tails transcriptome-wide in the context of PAPBN1 phosphorylation *in vivo*. poly(A) tail lengths in PABPN1 mutants were measured as follows: (**A**) northern blot of RNase T1-digested total RNA isolated from PABPN1 mutant cell lines treated with either DMSO or actinomycin D for 4 h prior to cell harvest. (**B**) Schematic of long-read sequencing library preparation strategy. Total RNA was isolated from PABPN1 mutant cells and reverse transcribed with TGIRT-III reverse transcriptase. A custom single-stranded DNA adapter was ligated to the 5′-end of cDNAs and the library was PCR amplified and sequenced using PacBio long-read sequencing. (**C**) Example of long reads from EV control RNA aligned to HNRNPA1 reference gene (top). Coverage and aligned long reads are shown in grey with soft-clipped poly(A) tails coloured orange. Reads displayed represent roughly 1.5% of reads mapping to HNRNPA1 in this sample. poly(A)+ short read coverage for the same condition is shown in blue. (**D**) Box plot of poly(A) tail lengths in EV, WT, 4SA and 4SD cells (two replicates combined). Median poly(A) tail lengths for each condition are indicated in each box. (**E**) Percent C, G and U in poly(A) tails for EV (green), WT (red), 4SA (blue) and 4SD (orange) at each nucleotide position with tails aligned at their 3′-ends. *P*-values (top red; dotted line indicates *P*= 0.05) for 4SA versus 4SD were calculated by two-tailed *t*-test.

We next investigated how the PABPN1 phosphorylation sites affected its ability to promote RNA polyadenylation. Because PAP is known to be inhibited by phosphorylation during mitosis (50), we over-expressed PABPN1 mutants in cycling cells (which are primarily in interphase, as mitosis is approximately 1 h of the 24 h cell cycle in human cells) to determine the effect of phosphorylation on PABPN1 alone, i.e. in the context of active PAP. We analyzed global poly(A) tail lengths from PABPN1 WT and mutant cells by northern blot (Figure 3A). This method involves incubating purified cellular RNA with RNase T1 to cleave all G residues, and therefore digests mRNAs but leaves their poly(A) tails intact and measurable (21). Under normal cellular conditions with active transcription, the 2SD and 4SD mutants had a similar distribution of poly(A) tail lengths as EV controls. In contrast, overexpressing WT, 2SA or 4SA PABPN1 resulted in marked poly(A) tail lengthening. To determine the maximum extent of hyperadenylation from each PABPN1 variant, we also analyzed poly(A) tails from cells treated for 2 h with transcription inhibitor Actinomycin D (Act D), which is known to result in PABPN1-dependent RNA hyperadenylation specifically in the nucleus (21). All of the PABPN1 WT and mutant cell lines were able to promote RNA hyperadenylation, but again the WT, 2SA and 4SA resulted in longer poly(A) tails than the 2SD and 4SD mutants (Figure 3A). Overall, the observation that 2SD and 4SD poly(A) tails are shorter suggests that phosphorylation of PABPN1 reduces polyadenylation activity and/or increases degradation of transcripts with long poly(A) tails.

To more precisely quantify the effect of the PABPN1 mutants on RNA polyadenylation, we prepared long-read sequencing (LRS) libraries for the PacBio Sequel II platform (51) using a strategy that simultaneously reports poly(A) tail length, poly(A) site selection and splicing status in single transcripts (Figure 3B). Our dataset contained 2 799 784 HiFi reads with median and maximum read lengths of 933 and 6 462 nucleotides, respectively, and 9 592 genes with greater than 100 reads per gene (Supplementary Figure S5A and Supplementary Figure S5B). We also prepared standard poly(A)+ Illumina RNA-seq libraries in parallel to assess gene expression (Figure 3C). We analyzed EV, WT, 4SA, and 4SD cells, as the quadruple mutants had the most substantial effects on polyadenylation. Consistent with our biochemical experiments, poly(A) tails were notably longer in 4SA cells compared to EV or 4SD cells (Figure 3D). The median poly(A) tail length for EV, WT, 4SA and 4SD cells was 86, 97, 108 and 87 nucleotides, respectively (two replicates combined) with good agreement between replicates (Supplementary Figure S5C). We conclude that poly(A) tail lengths are ∼20% longer in the transcriptome when PABPN1 phosphorylation is blocked.

The installation of non-A nucleotides in poly(A) tails is a known phenomenon that has been shown to affect RNA stability (2). We therefore aligned poly(A) tails at the cleavage site or the 3′-end and calculated the percent of each non-A nucleotide used at each position and compared non-A usage in tails between 4SA and 4SD mutants. Little difference in non-A usage was observed in tails aligned at the cleavage site (up to 400 nucleotides) (Supplementary Figure S5D). Interestingly, when we aligned tails at the 3′-end, we observed a decreased prevalence of non-A nucleotides in 4SA relative to EV and 4SD, particularly for C and U incorporation (Figure 3E). This suggests that phosphorylated PABPN1 may make RNAs vulnerable to modification by non-canonical RNA polymerases, possibly in the context of RNA degradation activity, and that such modification alters transcriptome homeostasis (52,53). We also examined APA between 4SA and 4SD from our Illumina sequencing dataset and found little evidence for PABPN1’s APA activity being altered by phosphorylation (Supplementary Figure S5E). Taken together, these findings suggest that the phosphorylation status of PABPN1 affects polyadenylation activity and results in altered incorporation of non-A nucleotides.

### Gene specificity of hyperadenylation by phospho-inhibitory PABPN1

PABPN1 is necessary for hyperadenylation and subsequent decay of several classes of nuclear RNAs, including antisense RNAs, intron-retained RNAs, and snoRNA host genes via PABPN1 and PAPα/γ-mediated decay (PPD) (21,54). This prompted us to investigate the specific targets of hyperadenylation by 4SA PABPN1. To directly compare poly(A) tail lengths per gene between conditions, we determined the distributions of tail lengths for each individual gene (two replicates combined). The distributions of lower quartile, median, and upper quartile poly(A) tail lengths were all longer in 4SA relative to 4SD cells, indicating that relative effects of the mutations were consistent across gene populations (Supplementary Figure S6A). Median poly(A) tail lengths per-gene were also consistent between replicates (Supplementary Figure S6B).

**Figure 4.**
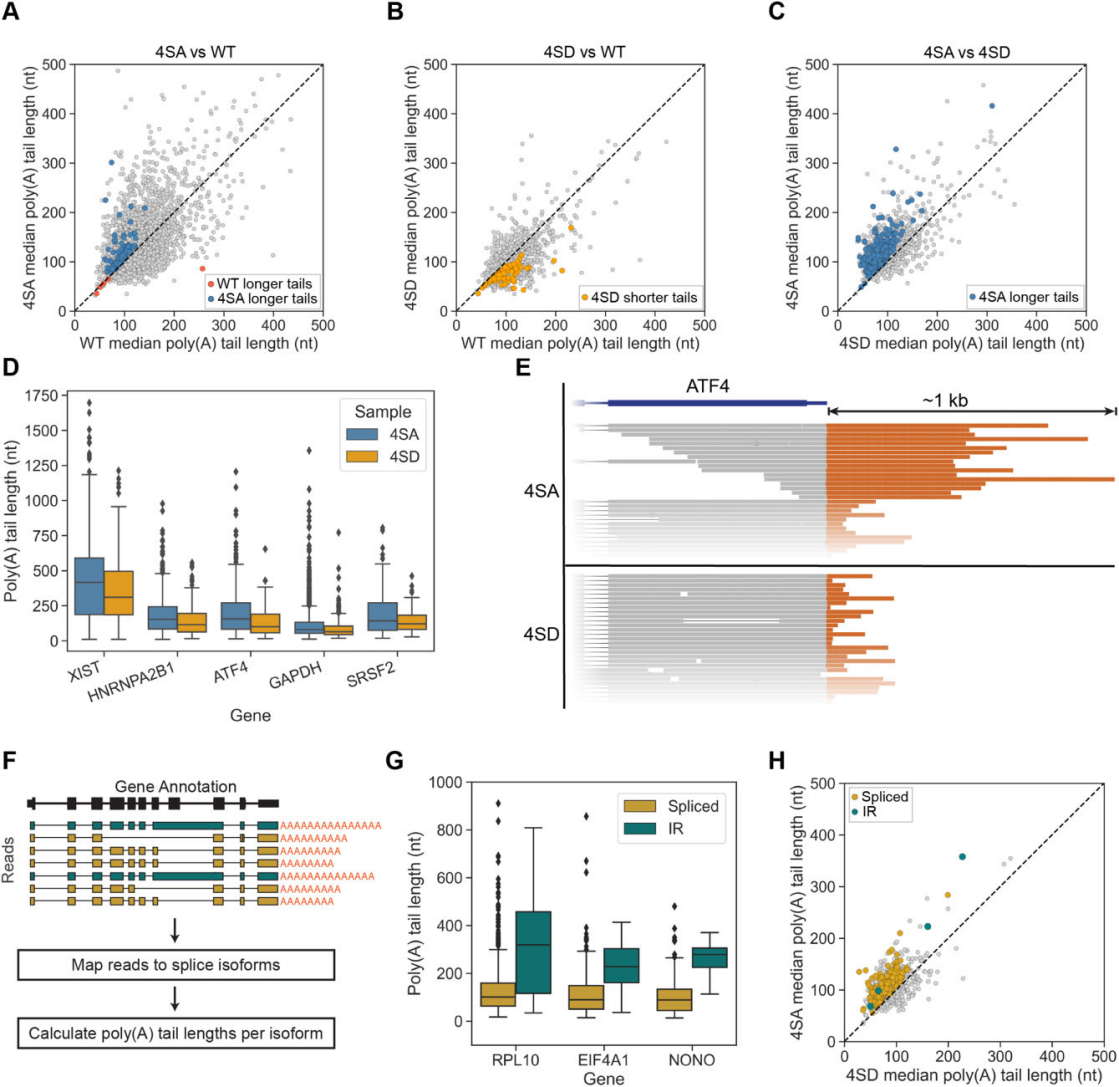
Phospho-inhibitory PABPN1 (4SA) mutants display widespread poly(A) tail lengthening of fully spliced and unspliced mRNA. (A–C) Scatterplot displaying median poly(A) tail length for each gene in (**A**) 4SA versus WT cells, (**B**) 4SD versus WT cells and (**C**) 4SA versus 4SD cells. Black dashed line represents equal tail lengths in both conditions. Genes whose transcripts have significantly longer poly(A) tails in one condition over the other are coloured accordingly as identified in the legend (see Figure 3D). Statistical significance was determined using Mann–Whitney *U* test and Benjamini–Hochberg correction (FDR < 0.05). (**D**) Boxplot of poly(A) tail lengths for reads mapped to select genes (two replicates combined) from 4SA and 4SD cells. (**E**) Schematic of poly(A) tail lengths in reads mapped to ATF4 in 4SA and 4SD cells. poly(A) tails are coloured orange. (**F**) Schematic of data analysis for poly(A) tail length comparisons between splice isoforms. (**G**) poly(A) tail lengths for RPL10, EIF4A1, and NONO spliced vs intron-retained isoforms in EV cells shows that intron-retained transcripts generally have longer poly(A) tails. (**H**) Scatterplot displaying median poly(A) tail length for each splice isoform in 4SA vs 4SD cells. Coloured points have significantly longer poly(A) tails in 4SA than 4SD cells. Gold points are fully spliced, while teal points are intron retained isoforms. Statistical significance was determined using Mann–Whitney *U* test and Benjamini–Hochberg correction (FDR < 0.05).

Because WT PABPN1 overexpression resulted in RNA hyperadenylation, we first compared 4SA and 4SD to WT cells. We found 104 genes that had had significantly longer and 5 genes that had significantly shorter poly(A) tails respectively in 4SA cells relative to WT cells (Figure 4A). In contrast, no genes displayed longer poly(A) tails in 4SD cells relative to WT cells (Figure 4B). Rather 95 genes had shorter poly(A) tails in 4SD relative to WT cells. We next compared 4SA directly to 4SD and found that 371 genes had significantly longer poly(A) tails in 4SA cells relative to 4SD cells but did not find any genes where the converse was true (Figure 4C). When we compared average read count to fold change in poly(A) tail lengths between 4SA and 4SD cells, the number of genes with significantly longer tails increased with read count (Supplementary Figure S6C), highlighting the possibility that the observed difference in poly(A) tail length is indeed transcriptome-wide, but highly expressed genes are more likely to be significant in our sequencing-based method. Taken together, our data suggest that phospho-inhibitory PABPN1 produces exaggerated polyadenylation activity relative to WT PABPN1, while polyadenylation activity is reduced for phospho-mimetic PABPN1.

Poly(A) tail lengths were highly variable within and between genes, and the 4SA mutant enhanced tail lengths differentially (see Figure 4C and D). The long noncoding RNA Xist exhibited the longest poly(A) tails in our dataset, consistent with previous reports (55), and the poly(A) tails were further lengthened in the 4SA mutant relative to 4SD (Figure 4D). Another example gene, ATF4, also showed dramatic increases in tail lengths (Figure 4E). To better determine the targets of 4SA PABPN1 with respect to splicing, we calculated poly(A) tail lengths per splice isoform (Figure 4F). In EV cells, intron-retained (IR) RNAs had longer poly(A) tails than their fully spliced counterparts in several compared genes (Figure 4G). 235 splice isoforms had longer poly(A) tails in 4SA cells than 4SD cells (Figure 4H). However, the majority (231) were fully spliced, indicating the 4SA PABPN1 does not favor IR transcripts shown previously to be targets of PPD. Rather, our results indicate that the poly(A) tail lengthening in the 4SA mutant is independent of RNA splicing status.

### PABPN1 mutants display transcriptome-wide alterations in RNA stability

PABPN1 is a central player in mRNA decay, both through its regulation of poly(A) tail lengths and its interaction with protein components of the RNA degradation machinery (7,8,11,56). Poly(A) tails play a pivotal role in tuning RNA stability, either protecting the RNAs from exonucleases or conversely marking them for degradation in the case of PABPN1-mediated hyperadenylation (21). Additionally, PABPN1 is a key component of the PAXT complex along with MTR4 and ZFC3H1, which targets nuclear poly(A)+ mRNA for degradation by the nuclear exosome (11). To first investigate whether phosphorylation of PABPN1 affected the levels of any previously identified PABPN1 mRNA targets, we first analyzed differential gene expression using our Illumina RNA-seq dataset. Overexpression of WT PABPN1 led to a relative decrease in the levels of antisense RNAs compared to EV control, consistent with increased PPD activity (Supplementary Figure S6D). Surprisingly few changes in relative RNA levels were detected between 4SA and 4SD cells. This prompted us to investigate whether RNA turnover could be altered by PABPN1 phosphorylation.

**Figure 5.**
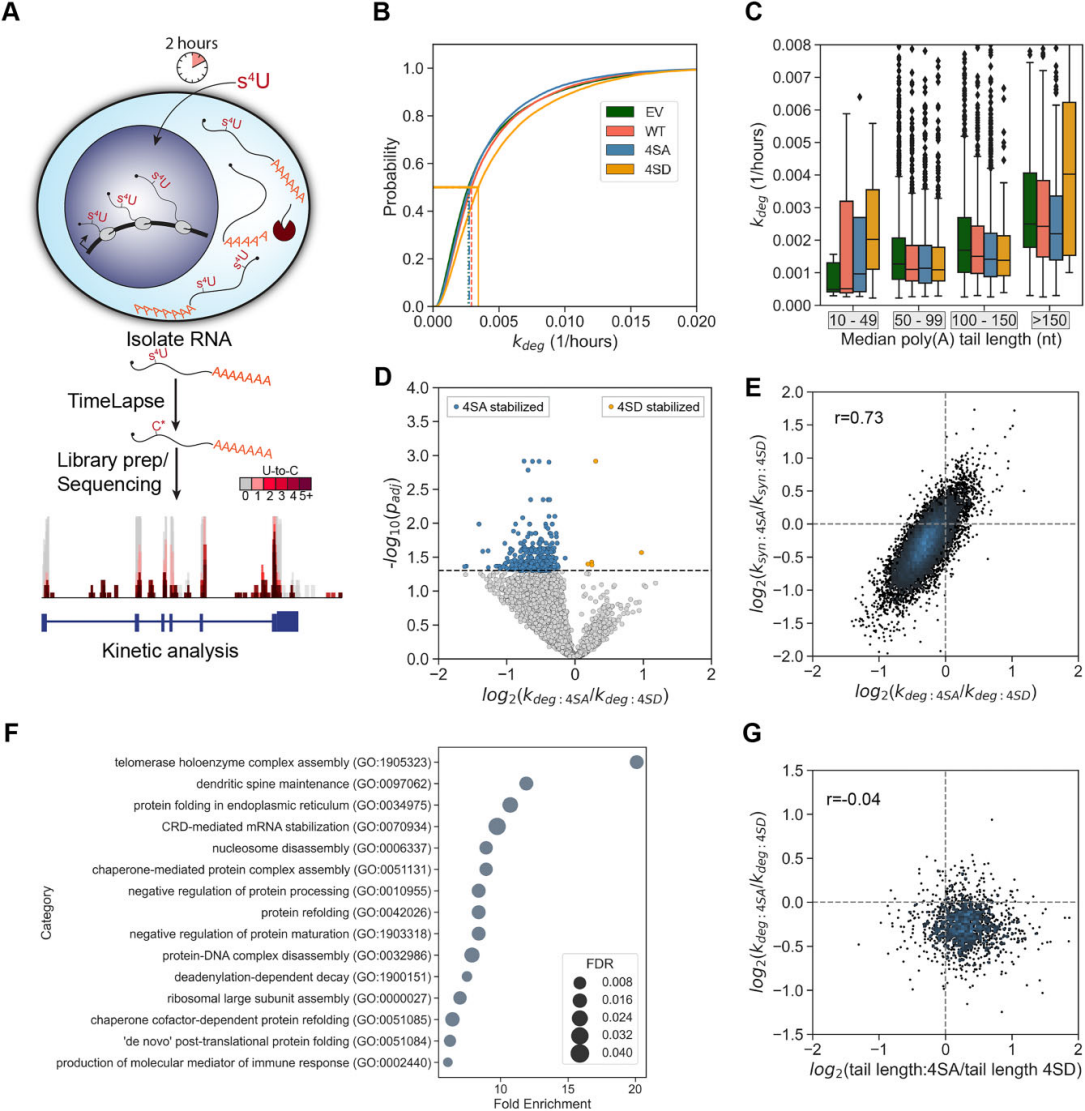
Phospho-mimetic PABPN1 (4SD) displays transcriptome-wide RNA destabilization. RNA stability was measured using TimeLapse-seq. (**A**) Schematic of TimeLapse-seq experiments. Colour scale (grey to maroon) indicates U-to-C mutations. (**B**) Cumulative distribution plot of *k_deg_* (1/h) for EV (dotted), WT (dashed), 4SA (dashed/dotted), and 4SD (solid) PABPN1. Median *k_deg_* is indicated. (**C**) *k_deg_* (1/h) for transcripts binned by median poly(A) tail length in PABPN1 mutants. (**D**) Volcano plot of log_2_(*k_deg_*_:_*_4SA_*/*k_deg_*_:_*_4SD_*). Transcripts with lower degradation rates in 4SA are coloured blue. Transcripts with lower degradation rates in 4SD are coloured orange. Significance cutoff was drawn at adjusted p-value of 0.05. 4SD was uses as the reference sample in data analysis. (**E**) Log_2_(*k_deg:4SA_*/*k_deg:4SD_*) versus Log_2_(*k_syn:4SA_*/*k_syn:4SD_*) (Pearson's correlation, *P*-value: <0.001, *r*: 0.73). (**F**) Top fifteen biological process GO enrichment terms for transcripts that are more stable in 4SA cells than 4SD cells. (**G**) Log_2_(tail length: 4SA/tail length:4SD) versus Log_2_(*k_deg:4SA_*/*k_deg:4SD_*) (Pearson's correlation, *P*-value: 0.09, *r*: −0.04).

To directly measure RNA turnover in the 4SA vs. 4SD mutants, we employed metabolic labeling-based TimeLapse sequencing (TimeLapse-seq), which can be used to infer degradation rate constants (*k*_deg_) and synthesis rate constants (*k*_syn_) of RNAs (Figure 5A, Supplementary Figure S7A, Supplementary Figure S7B) (28). We first compared global transcriptome stability between EV, WT, 4SA and 4SD cells (Figure 5B). The median *k_deg_* for EV, WT, 4SA and 4SD cells was 0.0027, 0.0029, 0.0028 and 0.0044, respectively, indicating that the 4SD PABPN1 caused a slight but significant global decrease in RNA stability relative to all other conditions. We next asked whether the observed differences in RNA stability between PABPN1 mutants were consistent across RNAs with varying median poly(A) tail lengths. When comparing stability of transcripts with different median tail lengths, *k_deg_* generally increased with median tail length in all conditions. Interestingly, the stability of transcripts with median poly(A) tails shorter than 50 nucleotides or longer than 150 nucleotides appeared to be disproportionately affected by mutations in the PABPN1 phosphorylation sites (Figure 5C). Thus, in addition to reducing polyadenylation, the phosphorylation status of PABPN1 may play an additional role in turnover of RNAs with short or long poly(A) tails.

Direct comparison of RNA stabilities between 4SA and 4SD cells revealed that many RNAs were markedly more stable in 4SA cells relative to 4SD (Figure 5D). Because we observed very few differences in RNA abundance between 4SA and 4SD cells, we wondered if *k_syn_* might be altered to offset changes in *k_deg_*. Indeed, many genes had significantly different *k_syn_* between 4SA and 4SD, with generally lower *k_syn_* in 4SA cells (Supplementary Figure S7C). Additionally, the log_2_ fold change (*k_deg_*) and log_2_ fold change (*k_syn_*) were highly correlated (Pearson's correlation coefficient 0.73, Figure 5E). Thus, despite general transcriptome instability, 4SD cells can maintain normal steady-state levels of RNA by turning up RNA synthesis.

**Figure 6.**
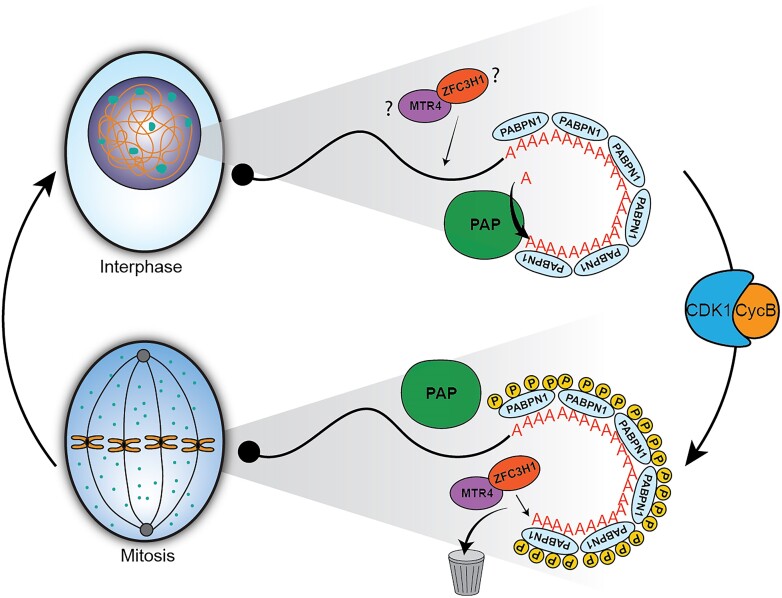
Working model: PABPN1 phosphorylation prevents hyperadenylation and maintains RNA dynamics. PABPN1 is phosphorylated in mitosis by CDK1-CyclinB, at which time it targets transcripts for degradation. This is likely due to continued interactions between phosphorylated PABPN1 and PAXT components. Prevention of PABPN1 phosphorylation leads to a transcriptome-wide poly(A) tail lengthening, aberrant stabilization of the dynamic transcriptome, and defective cellular proliferation.

We also sought to determine the identity of transcripts that were more stable upon phospho-inhibited PABPN1 expression compared to phospho-mimetic PABPN1. We analyzed the 387 4SA-stable transcripts for Gene Ontology enrichment. Interestingly, many stabilized transcripts encode proteins involved in RNA metabolism (Figure 5F), and several were involved in mitosis. For example, TOP2A mRNA was stabilized by 4SA PABPN1, and this mRNA is known to be rapidly degraded during mitotic exit (22) Thus, 4SA PABPN1 expression may hinder clearance of mitotic RNAs prior to cell division. To determine whether there was a direct relationship between poly(A) tail lengthening and RNA stability, we compared the log_2_ fold change in *k_deg_* to the log_2_ fold change log_2_ fold change in median poly(A) tail lengths between 4SA and 4SD (Figure 5G). Very little correlation was observed (Pearson's correlation coefficient −0.04), suggesting RNA stabilization by 4SA PABPN1 may be independent of the observed hyperadenylation. Altogether, our results support a model in which PABPN1 is phosphorylated in mitotic cells to dually prevent aberrant hyperadenylation and to target select mRNAs for degradation (Figure 6). We propose that phosphorylation of PABPN1 is essential to preserve the poly(A) tail lengths and dynamic stability of the transcriptome from mother cells to daughter cells.

## Discussion

Here, we describe a mechanism for tuning multiple activities of PABPN1 during the cell cycle. We demonstrate that PABPN1 is phosphorylated during mitosis in a CDK1-dependent manner, and that other kinases including DYRK3 may also participate. Mutations that prevent phosphorylation at these mitotic phosphorylation sites led to decreased cell proliferation, highlighting the biological importance of PABPN1 phosphorylation in human cells. We also show that phospho-mimetic and phospho-inhibitory mutants have specific effects on RNA poly(A) tail lengths and cause changes in RNA synthesis and stability, indicating tight control over mitotic transcriptomes by the polyadenylation machinery. Intriguingly, there is very little steady state difference among transcriptomes. Instead, our findings indicate that phosphorylation of PABPN1 increases transcriptome dynamics, by limiting poly(A) tail length and tuning mRNA turnover. Failure to phosphorylate PABPN1 leads to aberrant stabilization of mRNAs representing 387 genes, many of which are involved in cell growth and division. We propose phosphorylation of PABPN1 during mitosis leads to PABPN1-mediated turnover of RNA following mitotic exit. Below, we discuss this evidence and our working model that PABPN1-mediated mRNA turnover is an essential feature of cell proliferation (Figure 6).

What does phosphorylation do to PABPN1’s activity? In mitosis, phosphorylation of the nuclear poly(A) polymerase (PAP) reduces its polyadenylation activity (50). Perhaps decreased activity of PAP alone is not sufficient to prevent poly(A) tail extension during mitosis, particularly when stimulated by abundant unphosphorylated PABPN1. Overexpression of the phospho-mimetic PABPN1 4SD leads to tail lengths indistinguishable from EV control, while 4SA PABPN1 had significantly longer poly(A) tails than those expressing EV, WT or 4SD. We showed that WT, 4SA and 4SD PABPN1 can bind poly(A) RNA *in vitro*, making it unlikely that PABPN1 phosphorylation simply abrogates RNA binding. Indeed, 4SD and 4SA PABPN1 are both capable of interacting with other RNA-binding proteins in an RNA-dependent manner, indicating that both can bind RNA. If phosphorylated PABPN1 were completely inactive, we presume the 4SD would have dominant negative effects and poly(A) tails would be expected to shorten over a period of days. Because we did not observe this, we infer that phospho-PABPN1 must be at least partially active for poly(A) tail lengthening.

We speculate that PABPN1’s PAP-stimulating activity is likely reduced upon phosphorylation in mitosis, in concert with the inhibition of PAP. The four sites phosphorylated during mitosis are scattered throughout the PABPN1 molecule. Notably, PABPN1 uses its C-terminal 20 amino acids (287–306) as well as an internal alpha-helical domain (aa's 119–147) to stimulate PAP activity (7), and these regions are proximal to two of the mitotic phosphorylation sites we identified, S150 and S304. Mutation of each of these two sites to alanine was sufficient to induce RNA hyperadenylation and reduce cell proliferation. Thus, phosphorylation of PABPN1 on mitotic sites could feasibly reduce direct interactions with PAP or could alter a necessary conformational change in PABPN1 to carry out its stimulatory function.

Our findings agree with a recent report that mRNA stabilities are cell-cycle dependent (22). In that study, RNA-clearance from the mitosis-to-G1 transition were partially attributable to the cytoplasmic deadenylation factor CNOT1. Our work identifies PABPN1 as a nuclear factor that contributes to RNA turnover during mitosis when PABPN1 is phosphorylated. Thus, multiple PABPs may be required to maintain dynamic RNA turnover during various phases of the cell cycle. PABPN1 is a key regulator in the polyadenylation-dependent decay pathway of nuclear RNA (21,54). PABPN1 is involved in the PAXT connection with MTR4, and ZFC3H1 which recruits the nuclear exosome to nuclear polyadenylated RNAs (11,56). In support of this role, we show that expression of the phospho-mimetic PABPN1 mutant resulted in lower RNA stability compared to the phospho-inhibitory mutant. However, stability changes due to PABPN1 phosphorylation may not be directly coupled to poly(A) tail lengthening and may instead be due to transcript targeting to PAXT, because mRNA stability and median poly(A) tail lengths were not correlated on a per-gene basis. We therefore speculate that reduced PAP-stimulating activity might enhance PAXT-mediated decay, in the sense that free phospho-mimetic PABPN1 (i.e. PABPN1 that is not interacting with PAP or the cleavage machinery) is likely able to participate in the PAXT connection. Enhancing the PAXT connection over polyadenylation would lead to increased mRNA decay, either in the nucleus during interphase or in the mitotic plasm.

Another factor that could augment the phospho-PABPN1-mediated instability of mRNA could include the inclusion of non-A nucleotides in poly(A) tails that were induced upon overexpression of phospho-mimetic 4SD PABPN1. Non-canonical RNA polymerases, including TUTases, can install non-A nucleotides and affect stability of those RNAs (52). When we directly compared RNA stability in cells expressing 4SA versus 4SD PABPN1, the phospho-inhibitory protein 4SA resulted in higher RNA stability for many transcripts that code for proteins involved in replication. Thus, PABPN1 phosphorylation may be required to clear many mitosis-specific transcript from cells prior to cytokinesis or allowing for more dynamic regulation of mRNA stability by tuning poly(A) tail lengths and nucleotide composition as well as targeting specific RNAs for decay.

The discovery that PABPN1 is regulated by phosphorylation also raises the possibility that PABPN1 may be targeted by the same or different kinases in additional biological contexts. Note that roughly 20% of peptides mapping to S150 were phosphorylated in cycling cells (relative to 86% in mitotic cells, Figure 1D). These results suggest some level of phosphorylation during interphase at the sites identified in this study. During early embryonic development or under conditions of stress, mRNA polyadenylation and degradation are known to undergo dramatic changes (57). It is unknown if PABPN1 phosphorylation helps tune gene expression during early development. During early embryonic development especially, the cell cycle oscillates between S and M phases with no intervening G1 or G2 (58). Thus, PABPN1 phosphorylation may be required for precise control of poly(A) tail lengths and RNA stability during these transitions. It also is unknown whether PABPN1 is regulated by phosphorylation in terminally differentiated, non-mitotic cells such as neurons. Additional work will be required to uncover the breadth, importance, and regulatory potential of PABPN1 phosphorylation in different organisms and tissue types.

## Supplementary Material

gkae562_Supplemental_Files

## Data Availability

Raw and processed long-read sequencing, RNA-seq, and TimeLapse-seq data generated in this study are deposited in NCBI’s Gene Expression Omnibus and are accessible through GEO Series accession number GSE247007. Raw and processed proteomics data generated in this study are deposited in the PRIDE database under accession number PXD047110. All code used for LRS and TimeLapse-seq data analysis and for generating figures in this manuscript is available at https://github.com/NeugebauerLab/PABPN1_phosphorylation and https://zenodo.org/doi/10.5281/zenodo.11617988.

## Notes

Present address: David V. Phizicky, Waypoint Bio, Inc., 180 Varick Street, New York, NY 10014, USA.

## References

[1] Boreikaitė V. , Passmore L.A. 3′-End processing of eukaryotic mRNA: machinery, regulation, and impact on gene expression. Annu. Rev. Biochem. 2023; 92:199–225. 37001138 10.1146/annurev-biochem-052521-012445 PMC7614891

[2] Passmore L.A. , Coller J. Roles of mRNA poly(A) tails in regulation of eukaryotic gene expression. Nat. Rev. Mol. Cell Biol. 2022; 23:93–106. 34594027 10.1038/s41580-021-00417-y PMC7614307

[3] Gray N.K. , Coller J.M., Dickson K.S., Wickens M. Multiple portions of poly(A)-binding protein stimulate translation in vivo. EMBO J. 2000; 19:4723–4733. 10970864 10.1093/emboj/19.17.4723 PMC302064

[4] Bernstein P. , Peltz S.W., Ross J. The poly(A)-poly(A)-binding protein complex is a major determinant of mRNA stability in vitro. Mol. Cell. Biol. 1989; 9:659–670. 2565532 10.1128/mcb.9.2.659-670.1989 PMC362643

[5] Eichhorn S.W. , Subtelny A.O., Kronja I., Kwasnieski J.C., Orr-Weaver T.L., Bartel D.P. mRNA poly(A)-tail changes specified by deadenylation broadly reshape translation in Drosophila oocytes and early embryos. eLife. 2016; 5:e16955. 27474798 10.7554/eLife.16955 PMC4988829

[6] Lim J. , Lee M., Son A., Chang H., Kim V.N. mTAIL-seq reveals dynamic poly(A) tail regulation in oocyte-to-embryo development. Genes Dev. 2016; 30:1671–1682. 27445395 10.1101/gad.284802.116 PMC4973296

[7] Kerwitz Y. , Kühn U., Lilie H., Knoth A., Scheuermann T., Friedrich H., Schwarz E., Wahle E. Stimulation of poly(A) polymerase through a direct interaction with the nuclear poly(A) binding protein allosterically regulated by RNA. EMBO J. 2003; 22:3705–3714. 12853485 10.1093/emboj/cdg347 PMC165617

[8] Kühn U. , Gündel M., Knoth A., Kerwitz Y., Rüdel S., Wahle E. Poly(A) tail length is controlled by the nuclear poly(A)-binding protein regulating the interaction between poly(A) polymerase and the cleavage and polyadenylation specificity factor. J. Biol. Chem. 2009; 284:22803–22814. 19509282 10.1074/jbc.M109.018226 PMC2755688

[9] Fan X. , Dion P., Laganiere J., Brais B., Rouleau G.A. Oligomerization of polyalanine expanded PABPN1 facilitates nuclear protein aggregation that is associated with cell death. Hum. Mol. Genet. 2001; 10:2341–2351. 11689481 10.1093/hmg/10.21.2341

[10] Banerjee A. , Apponi L.H., Pavlath G.K., Corbett A.H. PABPN1: molecular function and muscle disease. FEBS J. 2013; 280:4230–4250. 23601051 10.1111/febs.12294 PMC3786098

[11] Meola N. , Domanski M., Karadoulama E., Chen Y., Gentil C., Pultz D., Vitting-Seerup K., Lykke-Andersen S., Andersen J.S., Sandelin A. et al. Identification of a nuclear exosome decay pathway for processed transcripts. Mol. Cell. 2016; 64:520–533. 27871484 10.1016/j.molcel.2016.09.025

[12] Jenal M. , Elkon R., Loayza-Puch F., van Haaften G., Kühn U., Menzies F.M., Oude Vrielink J.A., Bos A.J., Drost J., Rooijers K. et al. The poly(A)-binding protein nuclear 1 suppresses alternative cleavage and polyadenylation sites. Cell. 2012; 149:538–553. 22502866 10.1016/j.cell.2012.03.022

[13] Huang L. , Li G., Du C., Jia Y., Yang J., Fan W., Xu Y.Z., Cheng H., Zhou Y. The polyA tail facilitates splicing of last introns with weak 3′ splice sites via PABPN1. EMBO Rep. 2023; 24:e57128. 37661812 10.15252/embr.202357128 PMC10561182

[14] Kwiatek L. , Landry-Voyer A.M., Latour M., Yague-Sanz C., Bachand F. PABPN1 prevents the nuclear export of an unspliced RNA with a constitutive transport element and controls human gene expression via intron retention. RNA. 2023; 29:644–662. 36754576 10.1261/rna.079294.122 PMC10158996

[15] Gordon J.M. , Phizicky D.V., Neugebauer K.M. Nuclear mechanisms of gene expression control: pre-mRNA splicing as a life or death decision. Curr. Opin. Genet. Dev. 2021; 67:67–76. 33291060 10.1016/j.gde.2020.11.002 PMC8084925

[16] Krause S. , Fakan S., Weis K., Wahle E. Immunodetection of poly(A) binding protein II in the cell nucleus. Exp. Cell. Res. 1994; 214:75–82. 8082750 10.1006/excr.1994.1235

[17] Tavanez J.P. , Calado P., Braga J., Lafarga M., Carmo-Fonseca M. In vivo aggregation properties of the nuclear poly(A)-binding protein PABPN1. RNA. 2005; 11:752–762. 15811916 10.1261/rna.7217105 PMC1370760

[18] Galganski L. , Urbanek M.O., Krzyzosiak W.J. Nuclear speckles: molecular organization, biological function and role in disease. Nucleic Acids Res. 2017; 45:10350–10368. 28977640 10.1093/nar/gkx759 PMC5737799

[19] Palozola K.C. , Donahue G., Liu H., Grant G.R., Becker J.S., Cote A., Yu H., Raj A., Zaret K.S. Mitotic transcription and waves of gene reactivation during mitotic exit. Science. 2017; 358:119–122. 28912132 10.1126/science.aal4671 PMC5727891

[20] Park J.E. , Yi H., Kim Y., Chang H., Kim V.N. Regulation of poly(A) tail and translation during the somatic cell cycle. Mol. Cell. 2016; 62:462–471. 27153541 10.1016/j.molcel.2016.04.007

[21] Bresson S.M. , Hunter O.V., Hunter A.C., Conrad N.K. Canonical poly(A) polymerase activity promotes the decay of a wide variety of mammalian nuclear RNAs. PLoS Genet. 2015; 11:e1005610. 26484760 10.1371/journal.pgen.1005610 PMC4618350

[22] Krenning L. , Sonneveld S., Tanenbaum M.E. Time-resolved single-cell sequencing identifies multiple waves of mRNA decay during the mitosis-to-G1 phase transition. eLife. 2022; 11:e71356. 35103592 10.7554/eLife.71356 PMC8806192

[23] Schindelin J. , Arganda-Carreras I., Frise E., Kaynig V., Longair M., Pietzsch T., Preibisch S., Rueden C., Saalfeld S., Schmid B. et al. Fiji: an open-source platform for biological-image analysis. Nat. Methods. 2012; 9:676–682. 22743772 10.1038/nmeth.2019 PMC3855844

[24] Stringer C. , Wang T., Michaelos M., Pachitariu M. Cellpose: a generalist algorithm for cellular segmentation. Nat. Methods. 2021; 18:100–106. 33318659 10.1038/s41592-020-01018-x

[25] Gilbert W.V. , Zhou K., Butler T.K., Doudna J.A. Cap-independent translation is required for starvation-induced differentiation in yeast. Science. 2007; 317:1224–1227. 17761883 10.1126/science.1144467

[26] Torregrossa M.M. , MacDonald M., Stone K.L., Lam T.T., Nairn A.C., Taylor J.R. Phosphoproteomic analysis of cocaine memory extinction and reconsolidation in the nucleus accumbens. Psychopharmacology (Berl.). 2019; 236:531–543. 30411139 10.1007/s00213-018-5071-9 PMC6374162

[27] Feoktistova M. , Geserick P., Leverkus M. Crystal violet assay for determining viability of cultured cells. Cold Spring Harb. Protoc. 2016; 2016:pdb.prot087379. 27037069 10.1101/pdb.prot087379

[28] Schofield J.A. , Duffy E.E., Kiefer L., Sullivan M.C., Simon M.D. TimeLapse-seq: adding a temporal dimension to RNA sequencing through nucleoside recoding. Nat. Methods. 2018; 15:221–225. 29355846 10.1038/nmeth.4582 PMC5831505

[29] Martin M. Cutadapt removes adapter sequences from high-throughput sequencing reads. 2011. 2011; 17:3.

[30] Schmieder R. , Edwards R. Quality control and preprocessing of metagenomic datasets. Bioinformatics. 2011; 27:863–864. 21278185 10.1093/bioinformatics/btr026 PMC3051327

[31] Li H. Minimap2: pairwise alignment for nucleotide sequences. Bioinformatics. 2018; 34:3094–3100. 29750242 10.1093/bioinformatics/bty191 PMC6137996

[32] Li H. , Handsaker B., Wysoker A., Fennell T., Ruan J., Homer N., Marth G., Abecasis G., Durbin R. The sequence alignment/map format and SAMtools. Bioinformatics. 2009; 25:2078–2079. 19505943 10.1093/bioinformatics/btp352 PMC2723002

[33] Quinlan A.R. , Hall I.M. BEDTools: a flexible suite of utilities for comparing genomic features. Bioinformatics. 2010; 26:841–842. 20110278 10.1093/bioinformatics/btq033 PMC2832824

[34] Tang A.D. , Soulette C.M., van Baren M.J., Hart K., Hrabeta-Robinson E., Wu C.J., Brooks A.N. Full-length transcript characterization of SF3B1 mutation in chronic lymphocytic leukemia reveals downregulation of retained introns. Nat. Commun. 2020; 11:1438. 32188845 10.1038/s41467-020-15171-6 PMC7080807

[35] Mölder F. , Jablonski K.P., Letcher B., Hall M.B., Tomkins-Tinch C.H., Sochat V., Forster J., Lee S., Twardziok S.O., Kanitz A. et al. Sustainable data analysis with Snakemake. F1000Res. 2021; 10:33. 34035898 10.12688/f1000research.29032.1 PMC8114187

[36] Chen S. , Zhou Y., Chen Y., Gu J. fastp: an ultra-fast all-in-one FASTQ preprocessor. Bioinformatics. 2018; 34:i884–i890. 30423086 10.1093/bioinformatics/bty560 PMC6129281

[37] Dobin A. , Davis C.A., Schlesinger F., Drenkow J., Zaleski C., Jha S., Batut P., Chaisson M., Gingeras T.R. STAR: ultrafast universal RNA-seq aligner. Bioinformatics. 2013; 29:15–21. 23104886 10.1093/bioinformatics/bts635 PMC3530905

[38] Ha K.C.H. , Blencowe B.J., Morris Q. QAPA: a new method for the systematic analysis of alternative polyadenylation from RNA-seq data. Genome Biol. 2018; 19:45. 29592814 10.1186/s13059-018-1414-4 PMC5874996

[39] Love M.I. , Huber W., Anders S. Moderated estimation of fold change and dispersion for RNA-seq data with DESeq2. Genome Biol. 2014; 15:550. 25516281 10.1186/s13059-014-0550-8 PMC4302049

[40] Narasimhan V. , Danecek P., Scally A., Xue Y., Tyler-Smith C., Durbin R. BCFtools/RoH: a hidden Markov model approach for detecting autozygosity from next-generation sequencing data. Bioinformatics. 2016; 32:1749–1751. 26826718 10.1093/bioinformatics/btw044 PMC4892413

[41] Vock I.W. , Simon M.D. bakR: uncovering differential RNA synthesis and degradation kinetics transcriptome-wide with bayesian hierarchical modeling. RNA. 2023; 29:958–976. 37028916 10.1261/rna.079451.122 PMC10275263

[42] Gavish-Izakson M. , Velpula B.B., Elkon R., Prados-Carvajal R., Barnabas G.D., Ugalde A.P., Agami R., Geiger T., Huertas P., Ziv Y. et al. Nuclear poly(A)-binding protein 1 is an ATM target and essential for DNA double-strand break repair. Nucleic Acids Res. 2018; 46:730–747. 29253183 10.1093/nar/gkx1240 PMC5778506

[43] Pluta A.J. , Studniarek C., Murphy S., Norbury C.J. Cyclin-dependent kinases: masters of the eukaryotic universe. Wiley Interdiscip. Rev. RNA. 2023; 15:e1816. 37718413 10.1002/wrna.1816 PMC10909489

[44] Johnson J.L. , Yaron T.M., Huntsman E.M., Kerelsky A., Song J., Regev A., Lin T.Y., Liberatore K., Cizin D.M., Cohen B.M. et al. An atlas of substrate specificities for the human serine/threonine kinome. Nature. 2023; 613:759–766. 36631611 10.1038/s41586-022-05575-3 PMC9876800

[45] Rai A.K. , Chen J.X., Selbach M., Pelkmans L. Kinase-controlled phase transition of membraneless organelles in mitosis. Nature. 2018; 559:211–216. 29973724 10.1038/s41586-018-0279-8

[46] Sacco-Bubulya P. , Spector D.L. Disassembly of interchromatin granule clusters alters the coordination of transcription and pre-mRNA splicing. J. Cell Biol. 2002; 156:425–436. 11827980 10.1083/jcb.200107017 PMC2173333

[47] Marie-Josée Sasseville A. , Caron A.W, Bourget L., Klein A.F., Dicaire M.J., Rouleau G.A., Massie B., Langelier Y., Brais B The dynamism of PABPN1 nuclear inclusions during the cell cycle. Neurobiol. Dis. 2006; 23:621–629. 16860991 10.1016/j.nbd.2006.05.015

[48] Snead W.T. , Gladfelter A.S. The Control Centers of biomolecular phase separation: how membrane surfaces, PTMs, and active processes regulate condensation. Mol. Cell. 2019; 76:295–305. 31604601 10.1016/j.molcel.2019.09.016 PMC7173186

[49] Rio D.C. Filter-binding assay for analysis of RNA-protein interactions. Cold Spring Harb. Protoc. 2012; 2012:1078–1081. 23028069 10.1101/pdb.prot071449

[50] Colgan D.F. , Murthy K.G., Prives C., Manley J.L. Cell-cycle related regulation of poly(A) polymerase by phosphorylation. Nature. 1996; 384:282–285. 8918882 10.1038/384282a0

[51] McCarthy A. Third generation DNA sequencing: pacific biosciences’ single molecule real time technology. Chem. Biol. 2010; 17:675–676. 20659677 10.1016/j.chembiol.2010.07.004

[52] Lim J. , Ha M., Chang H., Kwon S.C., Simanshu D.K., Patel D.J., Kim V.N. Uridylation by TUT4 and TUT7 marks mRNA for degradation. Cell. 2014; 159:1365–1376. 25480299 10.1016/j.cell.2014.10.055 PMC4720960

[53] Lim J. , Kim D., Lee Y.S., Ha M., Lee M., Yeo J., Chang H., Song J., Ahn K., Kim V.N Mixed tailing by TENT4A and TENT4B shields mRNA from rapid deadenylation. Science. 2018; 361:701–704. 30026317 10.1126/science.aam5794

[54] Beaulieu Y.B. , Kleinman C.L., Landry-Voyer A.M., Majewski J., Bachand F. Polyadenylation-dependent control of long noncoding RNA expression by the poly(A)-binding protein nuclear 1. PLoS Genet. 2012; 8:e1003078. 23166521 10.1371/journal.pgen.1003078 PMC3499365

[55] Nicholson-Shaw A.L. , Kofman E.R., Yeo G.W., Pasquinelli A.E. Nuclear and cytoplasmic poly(A) binding proteins (PABPs) favor distinct transcripts and isoforms. Nucleic Acids Res. 2022; 50:4685–4702. 35438785 10.1093/nar/gkac263 PMC9071453

[56] Contreras X. , Depierre D., Akkawi C., Srbic M., Helsmoortel M., Nogaret M., LeHars M., Salifou K., Heurteau A., Cuvier O. et al. PAPγ associates with PAXT nuclear exosome to control the abundance of PROMPT ncRNAs. Nat. Commun. 2023; 14:6745. 37875486 10.1038/s41467-023-42620-9 PMC10598014

[57] Woo Y.M. , Kwak Y., Namkoong S., Kristjánsdóttir K., Lee S.H., Lee J.H., Kwak H. TED-Seq identifies the dynamics of poly(A) length during ER stress. Cell Rep. 2018; 24:3630–3641. 30257221 10.1016/j.celrep.2018.08.084

[58] Brantley S.E. , Di Talia S. Cell cycle control during early embryogenesis. Development. 2021; 148:dev193128. 34164654 10.1242/dev.193128 PMC8255047

