# Supplementary material for: Phosphorylation of the nuclear poly(A) binding protein (PABPN1) during mitosis protects mRNA from hyperadenylation and maintains transcriptome dynamics
Source: Nucleic Acids Res. 2024 Jun 29;52(16):9886–903. doi: 10.1093/nar/gkae562 (PMC11381358; doi:10.1093/nar/gkae562)
Supplement: gkae562_Supplemental_Files [file gkae562_supplemental_files.zip › Gordon_PABPN1_NAR_supplement_final_revised.pdf]

Supplemental Information for:

Phosphorylation of the nuclear poly(A) binding protein (PABPN1) during mitosis protects mRNA from hyperadenylation and maintains transcriptome dynamics.

AUTHORS

Jackson M. Gordon[1,†], David V. Phizicky[1,2,†], Leonard Schärfen[1], Courtney L. Brown[1], Dahyana Arias Escayola[1], Jean Kanyo[3], TuKiet T. Lam[3], Matthew D. Simon[1], Karla M. Neugebauer[1,*]

[1]Department of Molecular Biophysics and Biochemistry, Yale University, New Haven CT 06520, USA

[2]Current Address: Waypoint Bio, Inc., 180 Varick Street, New York, NY 10014, USA

[3]Keck MS & Proteomics Resource, Yale School of Medicine, New Haven, CT 06520, USA

* To whom correspondence should be addressed. Tel: (203) 785-4857; Email: karla.neugebauer@yale.edu

† Joint Authors

SUPPLEMENTAL FIGURES

Supplemental Figure 1. Identification of PABPN1 phosphorylation in HCT116, HeLa and HEK293 cells during mitosis and effects of kinase inhibitors.

Supplemental Figure 2. PABPN1 phospho-mutants display altered cell proliferation.

Supplemental Figure 3. PABPN1 phospho-mutants localize to the nucleus and are diffuse throughout the cell during mitosis.

Supplemental Figure 4. PABPN1 phosphorylation does inhibit poly(A) RNA binding.

Supplemental Figure 5. Library features for poly(A) tail sequencing.

Supplemental Figure 6. Poly(A) tail length changes per-gene in PABPN1 phospho-mutants.

Supplemental Figure 7. TimeLapse-seq reveals altered RNA stability between PABPN1 phospho-mutants.

SUPPLEMENTAL TABLES

Supplemental Table 1. Oligonucleotides used in this study.
Supplemental Table 2. RNA Sequencing and mapping statistics.
Supplemental Table 3. Mass spectrometry of PABPN1-mCherry from interphase and mitotic cells (Excel spreadsheet).
Supplemental Table 4. Mass spectrometry of recombinant PABPN1 treated with CDK1 or DYRK3 kinase (Excel spreadsheet)
Supplemental Table 5. Mass spectrometry of PABPN1 mutant pull downs (Excel spreadsheet).

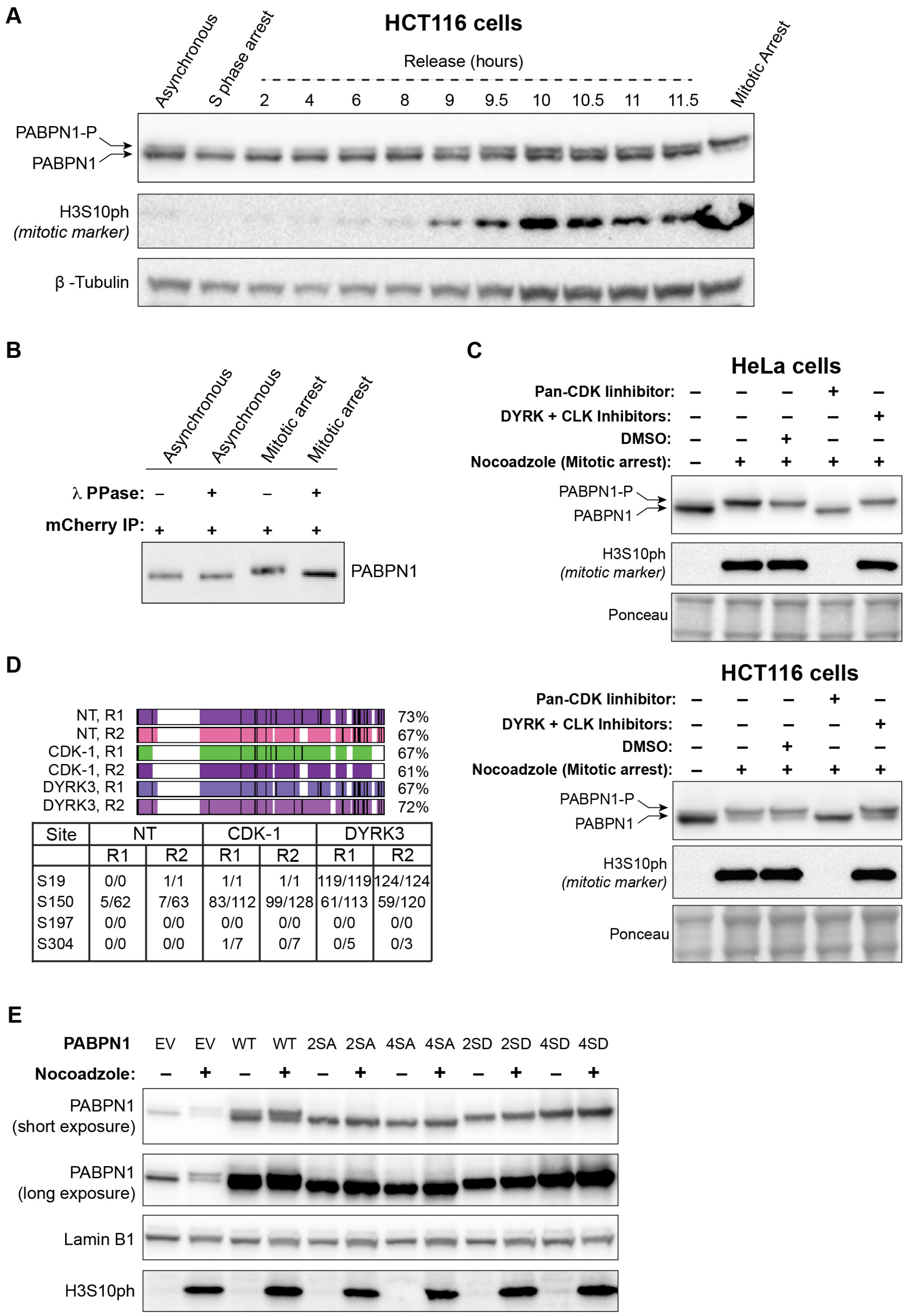

Supplemental Figure 1. Identification of PABPN1 phosphorylation in HCT116, HeLa and HEK293 cells during mitosis and effects of kinase inhibitors. (A) Anti-PABPN1 and anti-H3S10ph western blots of extracts of HCT116 cells arrested in S-phase or released into mitosis. Anti-β1 Tubulin was used to assess loading. (B) Anti-PABPN1 western blot of mCherry-PABPN1 immunoprecipitated from asynchronous HeLa cells or HeLa cells arrested with Nocodazole treatment and treated with lambda phosphatase. (C) Anti-PABPN1 and anti-H3S10ph western blots of extracts from HeLa cells (Top) or HCT116 cells (Bottom) treated with Pan-CDK inhibitor roscovitine or DYRK/CLK inhibitor cocktail. Ponceau was used to assess loading. (D) Mass spectrometry coverage of PABPN1 sequence for untreated recombinant PABPN1 (NT) or PABPN1 following kinase reactions containing purified CDK1-CyclinB (CDK-1) or MBP-DYRK3-His (DYRK3). Coverage for individual replicates is shown (Top). Corresponding modification and peptide count for S19, S150, S197, and S304 are displayed in the table (bottom). Individual replicates are shown. (E) Anti-PABPN1 and anti-H3S10ph western blots of extracts of HEK293 phospho-mutant expressing cells (see Fig 2 for details of the mutations) in interphase or arrested in mitosis with nocodazole. Anti-Lamin B1 was used to assess loading.

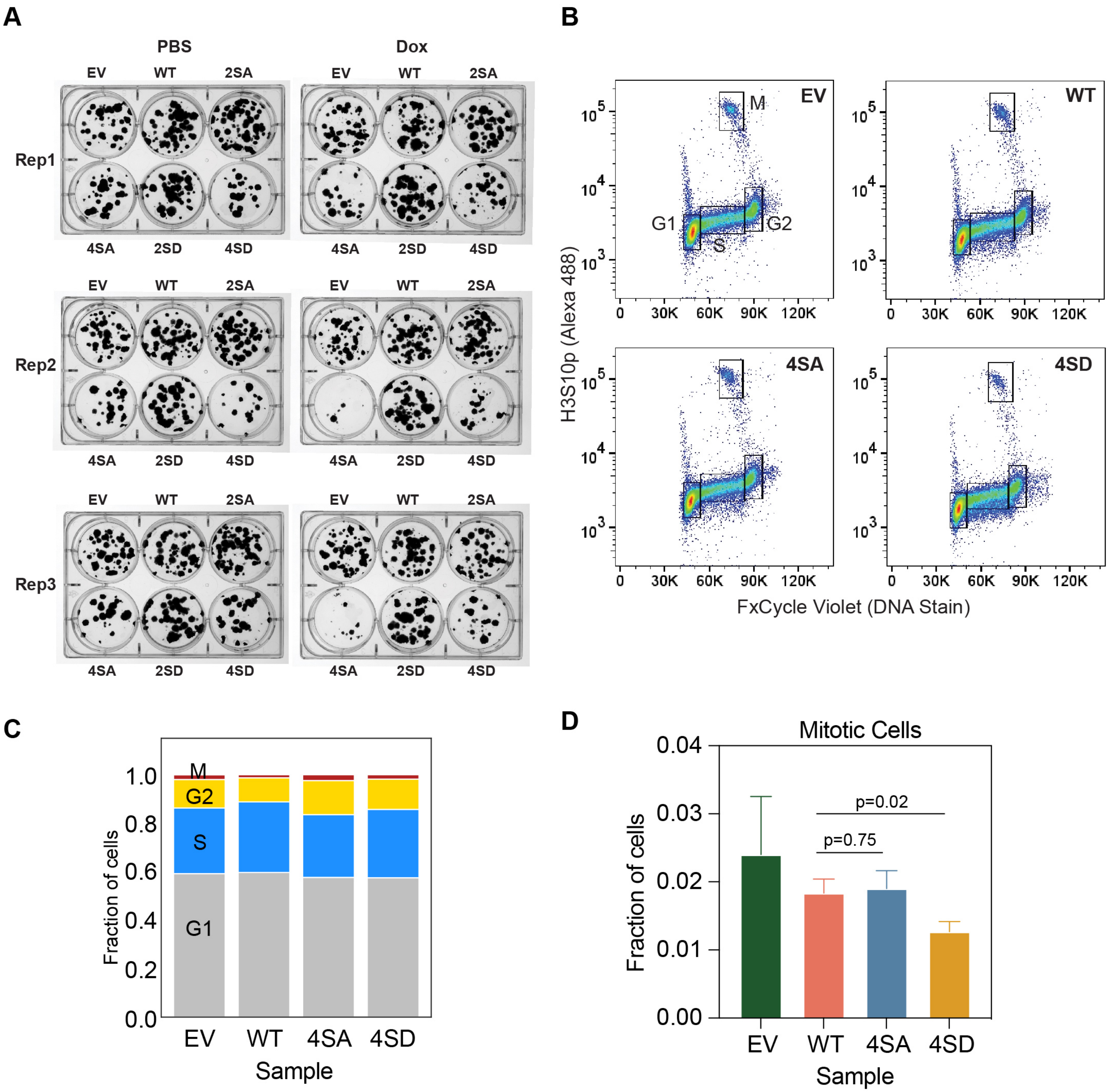

Supplemental Figure 2. PABPN1 phospho-mutants display altered cell proliferation. (A) Six-well plates containing crystal violet-stained cells from each PABPN1 mutant cell line grown in the presence of doxycycline or PBS or 14 days. Individual replicates are shown. (B) Cell cycle analysis by flow cytometry of PABPN1 phospho-mutants. (C) Quantification of mean fraction of cells in G1, S, G2, or M phase in PABPN1 phospho-mutants from cell cycle analysis. (D) Fraction of cells in mitosis for PABPN1 phospho-mutants. Two-tailed t-test was used for statistical testing between conditions.

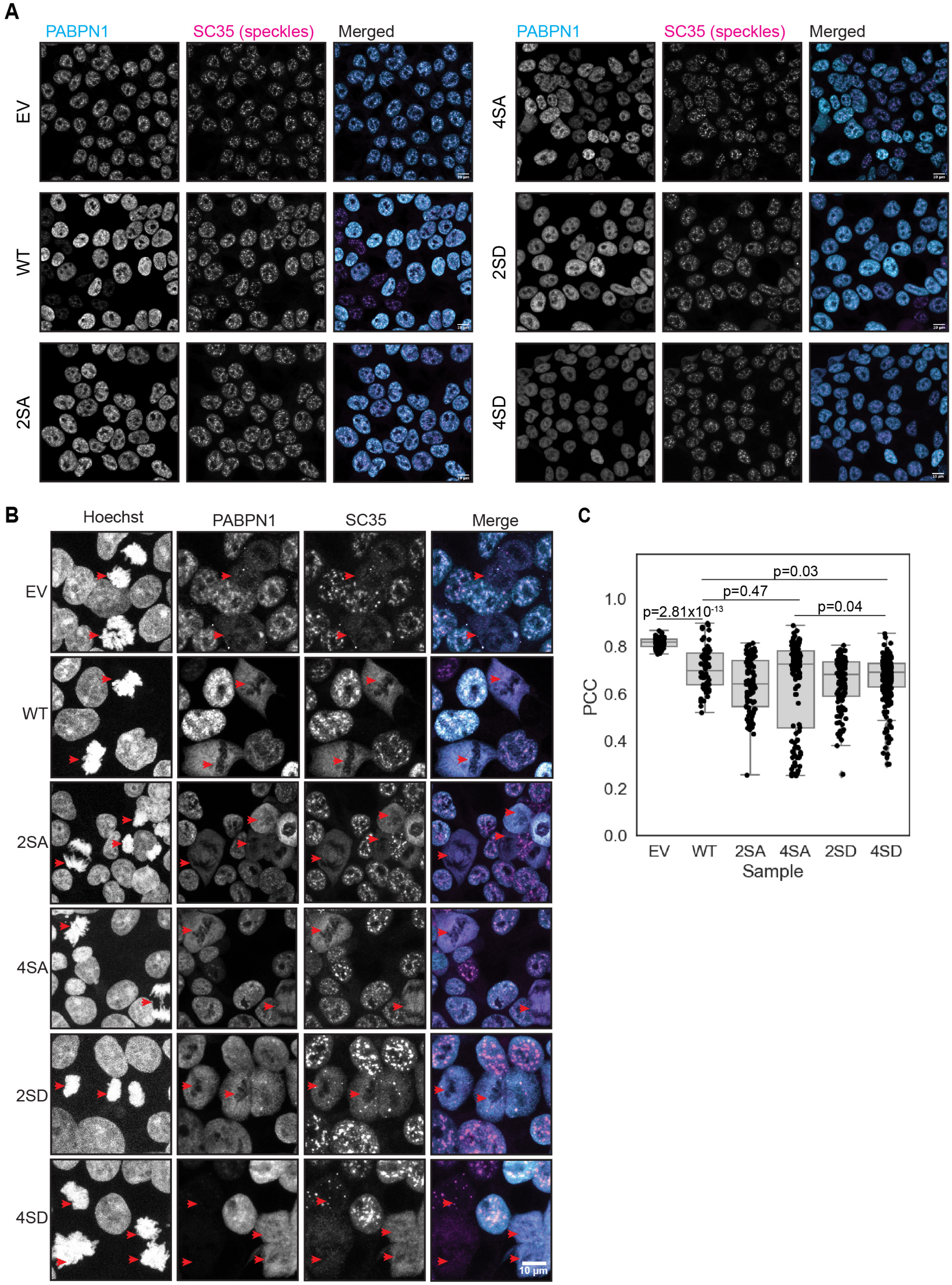

Supplemental Figure 3. PABPN1 phospho-mutants localize to the nucleus and are diffuse throughout the cell during mitosis. (A) Immunofluorescence microscopy images of PABPN1 phospho-mutant cell lines using anti-PABPN1 (left) and anti-SC35 (middle) antibodies. Merged images are shown on the right (PABPN1 in cyan, SC35 in magenta). (B) Zoom-ins of Immunofluorescence microscopy images of PABPN1 phospho-mutant with mitotic cells marked as red arrows. (C) Quantification of overlap (Pearson's correlation coefficient, PCC) between SC35 and PABPN1 from single nuclei in immunofluorescence microscopy images. Mann-Whitney U test was used for statistical testing between conditions.

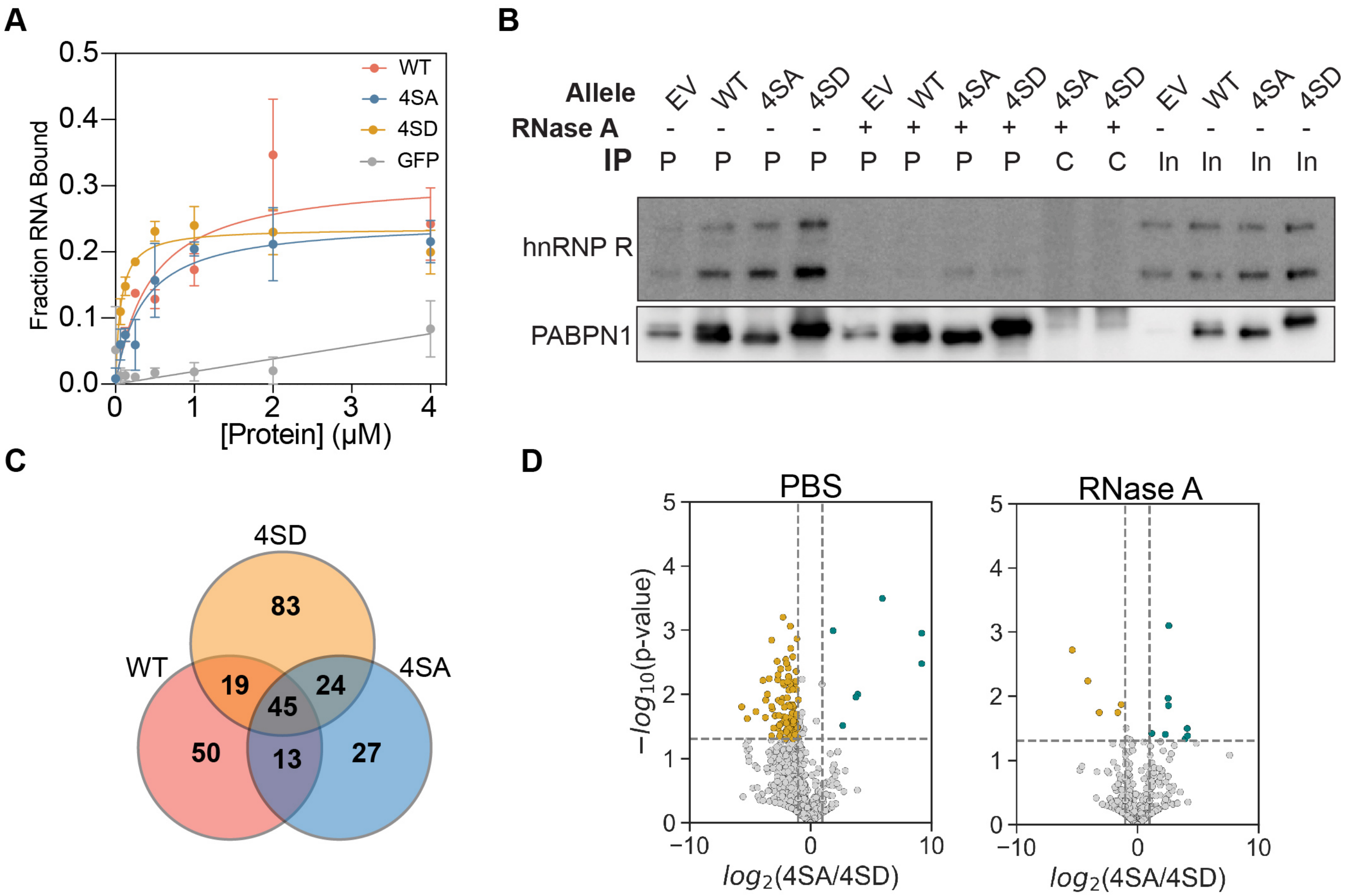

Supplemental Figure 4. PABPN1 phosphorylation does inhibit poly(A) RNA binding. (A) Binding curves for filter binding assays of WT, 4SA, or 4SD His-MBP-PABPN1 bound to poly(A)$_{30}$ RNA. GFP was used as a negative control. (B) anti-hnRNP R and anti-PABPN1 western blots following anti-PABPN1 Co-IPs. IP indicates antibody used for pulldown (P:anti-PABPN1, C:IgG control, I:Input) (C) Overlapping proteins that co-immunoprecipitated with WT, 4SA, or 4SD PABPN1, enriched in PBS treated samples over RNase A-treated samples (D) Volcano plots of proteins that co-immunoprecipitated 4SA (teal) over 4SD PABPN1 (teal), treated with PBS (left) or RNase A (right) and identified by mass spectrometry. Significance cutoff was drawn at $p < 0.05$.

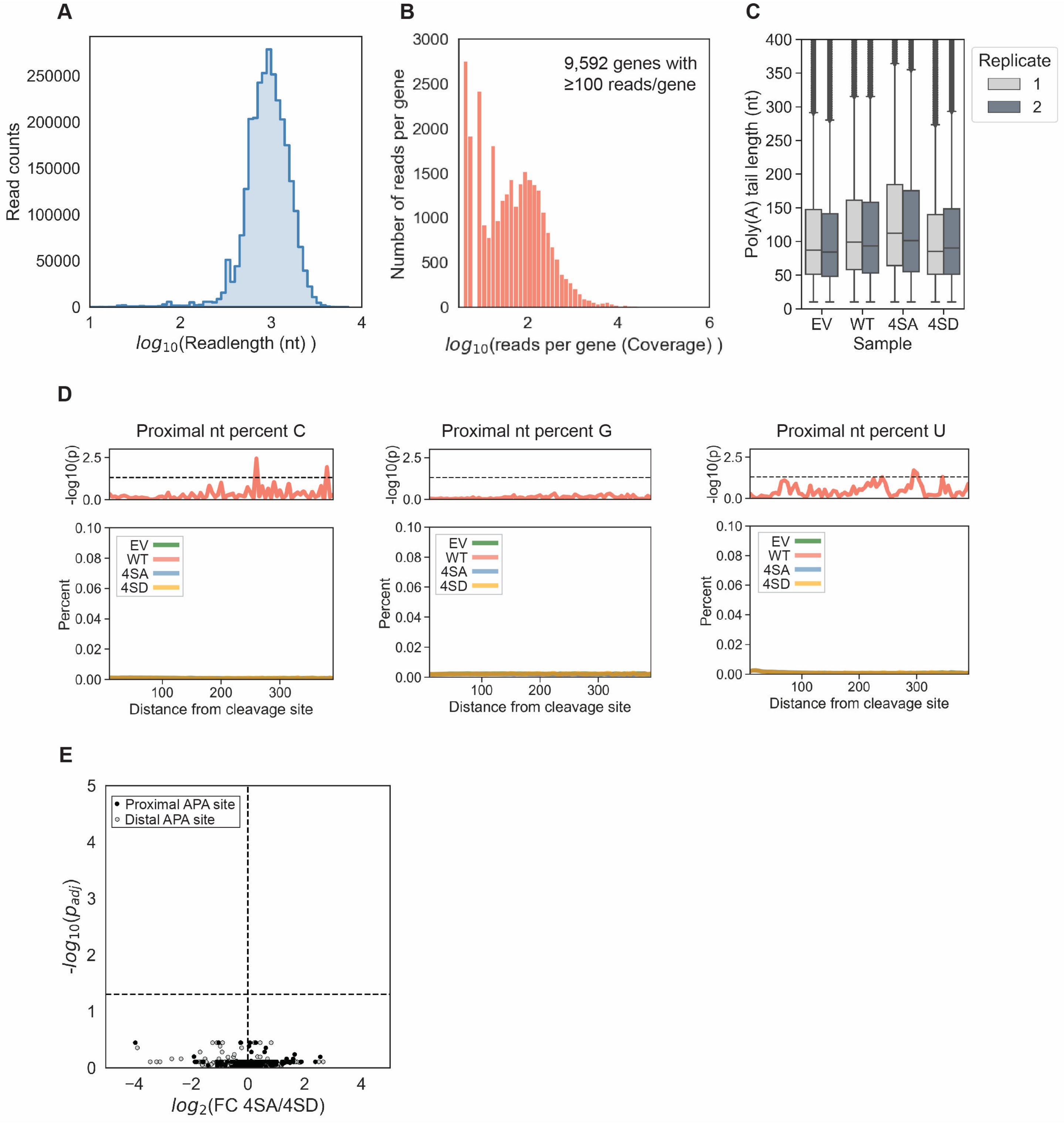

Supplemental Figure 5. Library features for poly(A) tail sequencing. (A) Read length distribution histogram for long-read sequencing dataset (all replicates and conditions combined). (B) Histogram of read coverage per gene for long-read sequencing dataset. (C) Boxplot of poly(A) tail lengths per condition for two biological replicates, calculated from long-read sequencing dataset. (D) Percent C, G, and U in poly(A) tails for EV (green), WT (Red), 4SA (blue) and 4SD (orange) at each nucleotide position with tails aligned at their cleavage site. P-values (top red; dotted line indicates p=0.05) for 4SA vs 4SD were calculated by two-tailed t-test. (E) Alternative polyadenylation site usage between 4SA and 4SD from Illumina sequencing data (dotted line indicates p_{adj}=0.05).

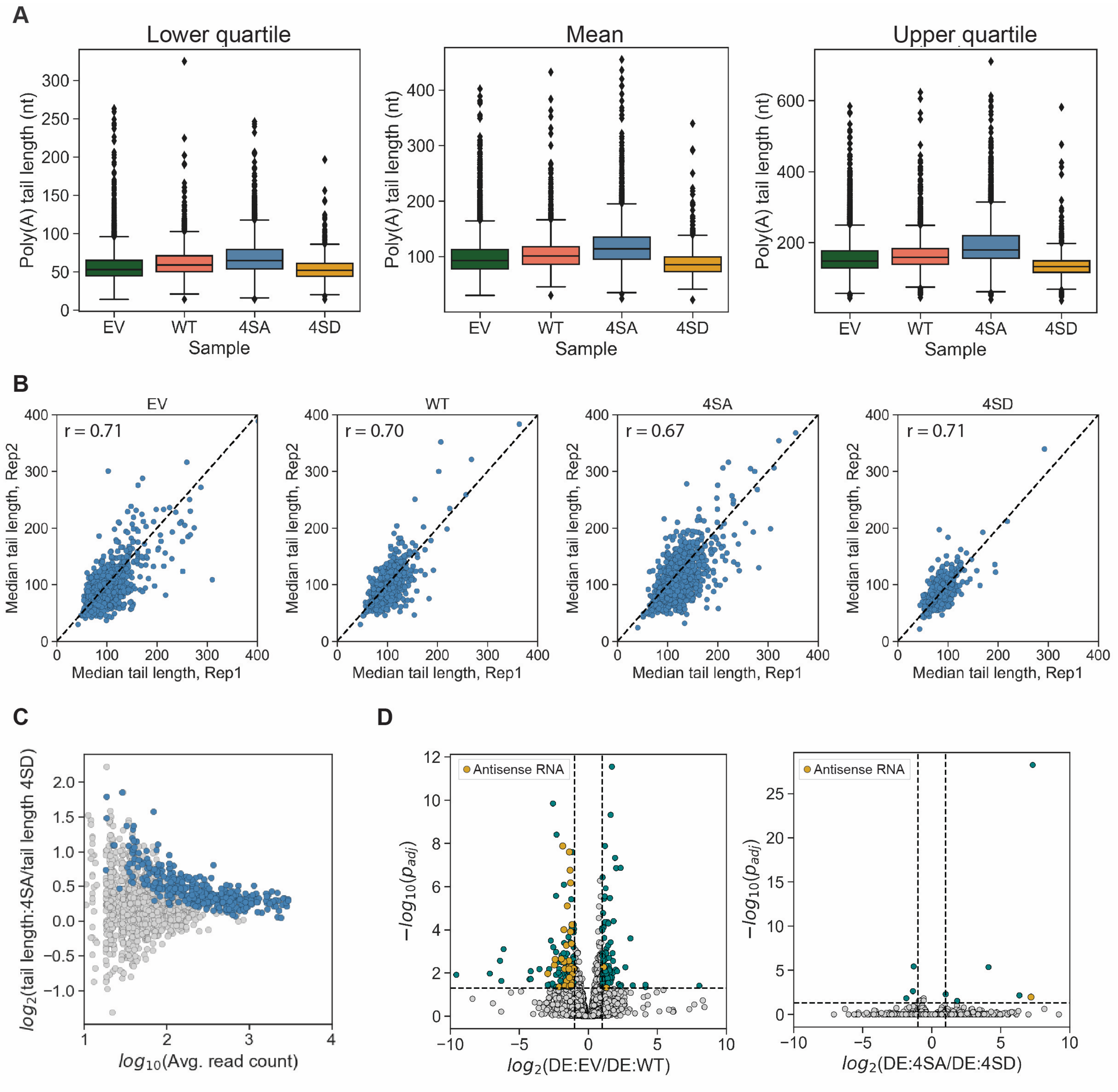

Supplemental Figure 6. Poly(A) tail length changes per-gene in PABPN1 phospho-mutants. (A) Boxplots for median, lower quartile, and upper quartile of poly(A) tail length distributions in each condition. (B) Correlation of median poly(A) tail lengths per gene between two biological replicates, separated by condition. Pearson's correlation coefficient for each comparison is shown. (C) Log_{10}(Average read count) vs. log_2(tail length:4SA/tail length:4SD). Genes with significantly longer poly(A) tails in 4SA are coloured blue. (D) Volcano plots of differentially expressed genes, determined by RNA-seq, for EV/WT (left) and 4SA/4SD PABPN1 (right). Significant non-antisense transcripts are coloured teal. Significant antisense transcripts are coloured gold.

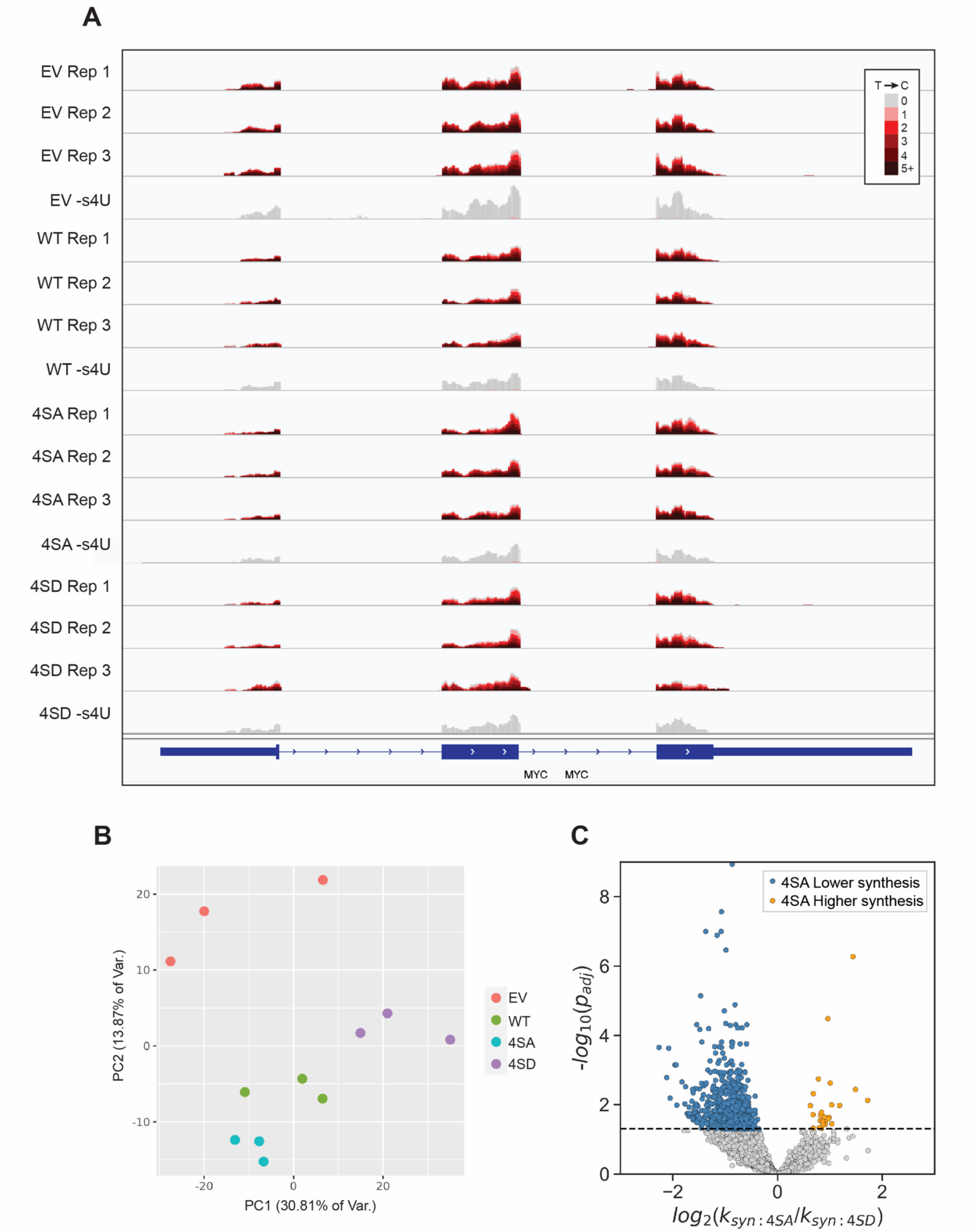

Supplemental Figure 7. TimeLapse-seq reveals altered RNA stability between PABPN1 phospho-mutants. (A) Genome browser shot of read coverage of MYC, coloured by number of T to C mutations. Read pileup scale is 0-295 for all samples. (B) Principal component analysis plot of mutation rates from TimeLapse-seq dataset. (C) Volcano plot of $\log_2(k_{syn:4SA}/k_{syn:4SD})$. Transcripts with lower synthesis rates in 4SA are coloured blue. Transcripts with higher synthesis rates in 4SA are coloured orange. Significance cutoff was drawn at p_{adj}=0.05.

Supplemental Table 1. Oligonucleotides used in this study

Description	Sequence
TGIRT 3′ Adapter RNA	rArArGrArUrCrGrGrArArGrArGrCrArCrArCrGrUrCrUrGrArArCrUrCrC rArGrUrCrArC/3SpC3/
TGIRT 3′ Adapter DNA	GTGACTGGAGTTCAGACGTGTGCTCTTCCGATCTTN
TGIRT 5′ Adapter DNA	/5Phos/GATCGTCGGACTGTAGAACTCTGAACGTGTAG/3SpC3/
PacBio Library PCR, Forward primer	GACGTGTGCTCTTCCGATCTACACACTCTATCAGATCTACACGT TCAGAGTTCTACAGTCCGACGATC
PacBio Library PCR, EV Reverse Primer	AAGCAGTGGTATCAACGCAGAGTACCACATATCAGAGTGCGGT GACTGGAGTTCAGACGTGTGCTCTTCCGATCT
PacBio Library PCR, WT Reverse Primer	AAGCAGTGGTATCAACGCAGAGTACACACACAGACTGTGAGGT GACTGGAGTTCAGACGTGTGCTCTTCCGATCT
PacBio Library PCR, 4SA Reverse Primer	AAGCAGTGGTATCAACGCAGAGTACACACATCTCGTGAGAGGT GACTGGAGTTCAGACGTGTGCTCTTCCGATCT
PacBio Library PCR, 4SD Reverse Primer	AAGCAGTGGTATCAACGCAGAGTACCACGCACACACGCGCGGT GACTGGAGTTCAGACGTGTGCTCTTCCGATCT
Oligo dT_{40} for northern blots	TTTTTTTTTTTTTTTTTTTTTTTTTTTTTTTTTTTTTTTT
Oligo A_{30} for filter binding assays.	rArArArArArArArArArArArArArArArArArArArArArArArArArArArArArA

Supplemental Table 2. RNA Sequencing and mapping statistics

Sample	Replicate	Platform	Uniquely mapped reads (hg38)	Median Read length (nt)	Number of polyadenylated reads
EV	1	PacBio	718631	908	532227
EV	2	PacBio	334473	1046	280775
WT	1	PacBio	496932	875	354934
WT	2	PacBio	167833	970	136709
4SA	1	PacBio	532471	894	392544
4SA	2	PacBio	268795	1024	224156
4SD	1	PacBio	141147	883	97377
4SD	2	PacBio	121039	990	99865
EV	1	NovaSeq	32983978	151	NA
EV	2	NovaSeq	33221775	151	NA
EV	3	NovaSeq	31817772	151	NA
WT	1	NovaSeq	33918630	151	NA
WT	2	NovaSeq	40933809	151	NA
WT	3	NovaSeq	32700731	151	NA
4SA	1	NovaSeq	40403051	151	NA
4SA	2	NovaSeq	34039730	151	NA
4SA	3	NovaSeq	29769305	151	NA
4SD	1	NovaSeq	32018748	151	NA
4SD	2	NovaSeq	31119941	151	NA
4SD	3	NovaSeq	31146534	151	NA
EV (TL)	1	NovaSeq	76175884	151	NA
EV (TL)	2	NovaSeq	72975648	151	NA
EV (TL)	3	NovaSeq	40183652	151	NA
EV (TL)	No s4U	NovaSeq	16957484	151	NA
WT (TL)	1	NovaSeq	47077644	151	NA
WT (TL)	2	NovaSeq	51821744	151	NA
WT (TL)	3	NovaSeq	26935968	151	NA
WT (TL)	No s4U	NovaSeq	60011264	151	NA
4SA (TL)	1	NovaSeq	82137450	151	NA
4SA (TL)	2	NovaSeq	70551948	151	NA
4SA (TL)	3	NovaSeq	53613422	151	NA
4SA (TL)	No s4U	NovaSeq	52714214	151	NA
4SD (TL)	1	NovaSeq	61174830	151	NA
4SD (TL)	2	NovaSeq	84315966	151	NA
4SD (TL)	3	NovaSeq	19566020	151	NA
4SD (TL)	No s4U	NovaSeq	62327046	151	NA
